# Traditional knowledge of wild plants on traditional tools, materials, products and economic practices in southern Yemen

**DOI:** 10.1186/s13002-024-00698-5

**Published:** 2024-06-19

**Authors:** Mohamed Al-Fatimi

**Affiliations:** Department of Pharmacognosy, Faculty of Pharmacy, University of Aden, Aden, Yemen

**Keywords:** Agriculture, Domestic, Ethnobotany, Handicraft, Southern Yemen

## Abstract

**Background:**

The traditional knowledge in southern Yemen is rich in wild medicinal and food plants, which has been documented in our previous studies. In addition, other significant and general traditional usage for the daily livelihood requirements of local people (beyond medicinal and food plant uses) has not been studied before and needs urgent documentation.

**Methods:**

Ethnobotanical data on of wild plants used by local people in southern Yemen were collected by oral questionnaire interviews. Most informants (n = 1020) were local elderly from 15 different localities in southern Yemen. The local names and non-medicinal and non-food uses of plants were identified and analyzed.

**Results:**

The ethnobotanical data resulted various traditional uses of 73 plant species distributed in 28 families. The most represented families were Fabaceae, Asteraceae and Malvaceae. The most growth forms were trees and shrubs. Seven main and common categories of traditional uses were determined and classified as handicraft, health aids, livestock husbandry and beekeeping, economic and commercial plant products, agriculture tools, construction timber and fuel. The most cited species were identified for *Ziziphus spina-christi, Vachellia tortilis, Vachellia nilotica*, *Anisotes trisulcus, Dracaena hanningtonii* (*Sansevieria ehrenbergii*) and *Aerva javanica*, which have multi-purpose values of traditional usage. Nine major traditional uses of local wild plants were recorded: handicraft, agriculture tools, products aid general health, economic products, construction timber, livestock husbandry, bee keeping, fuel and ornamental.

**Conclusions:**

Despite the challenges on local traditional knowledge of wild plants, it still requested vital to many usages of traditional life and still have an economic value and heritage required of develop the daily livelihood level of the local people especially in rural areas. This includes the traditional uses of wild plants in handicraft skills, tools of agriculture, constructions. The importance of the continuity of traditional industries and their transmission to generations lies in the local population’s reliance on local natural resources without relying on external resources in situations such as wars. This is the first study that contributes to documenting and analyzing the indigenous knowledge on traditional general usage of wild plants in southern Yemen.

## Introduction

Yemen is a unique country in the traditional heritage in the traditional uses of local plants in its daily life since ancient times, whether in nutrition, phytotherapy, or in other daily needs of human life. On the other hand, the flora of Yemen has not yet been fully explored, and many new plant species are still being discovered [1–3].

In this perspective, the local communities in northern Yemen [4–6] and southern Yemen [3, 7, 8] are rich in traditional knowledge of medicinal and edible wild plants. Moreover, this traditional knowledge is rich in wild plants with non-medicinal and non-food usage in various fields in the making of traditional tools of agricultural, handicrafts, domestics and cosmetic [9–11].

The ancient civilizations before 3000 years BC, passed through southern Yemen such as the kingdoms of Hadramout and Awsan and through northern Yemen such as kingdom of Saba in northern Yemen, must have had an impact on the strength of interdependence and relationship between tribes, plants and high knowledge in their inherited uses. Yemen in general has the most important agricultural heritage and traditions among the countries of the Arab peninsula, where centuries before AD, they used the stars as agricultural markers [12]. Many ancient Arab manuscripts from more than a thousand years ago documented the historical development and experiences of the local population in Yemen in agriculture in the mountains and valleys and the use of tools to develop agriculture for more and better agricultural production; moreover, they possessed a traditional skill, the ability to overcome diseases that affect crops [9].

Trade was active between the ancient pre-Islamic Arab kingdoms and the countries of Africa and Asia, which helped introduce new knowledge of agriculture and introduce new crops into agriculture, such as *Triticum* sp., *Hordeum* sp, coffee from Africa and *Gossypium* sp. and *Sesamum indicum* from south Asia [13]. Therefore, since ancient Kingdoms in southern Yemen were famous for traditional agriculture including of different crops such as wheat, corn, sorghum, millet and coffee, using traditional agricultural tools made from local plants [10]. Today, in southern Yemen, there are six variants of *Coffea arabica* growing in the districts of Yafee, Rusud and Al-Dhalee [14].

Thousands of years ago, aloe and frankincense have been the most famous plant products in the global trade from southern Yemen, Socotra and Oman [15], followed by gums, dyes and myrrh, as well as the use of tree wood in the construction of doors, windows and roofs of houses and palaces [16], some of these buildings still exist from a thousand to 500 to years ago such skyscraper buildings made of clay and wood which still exists today in Hadhramout for more than five centuries. During the history, the inherited practices of the local people in southern Yemen have developed in the ability to process many traditional tools from wood and stems of plants to help them in daily life, which is not devoid of creations that indicate inherited popular awareness and the ability to think to create useful and economic material from the nature to develop the standard of life.

There is a lack of comprehensive field studies published on Arab countries on all traditional uses of plants from the daily needs of the home to agricultural and construction tools, except for some of the uses reported from Oman [17]. This is due to the similarity of flora and environmental, ethnic, historical and cultural factors between southern Yemen and Oman. This applies to all countries of the Arabian Peninsula, and therefore, it is expected that there will be a great similarity in the ethnobotany of these countries and perhaps this traditional knowledge from Sothern Yemen circulates among people throughout the Arabian world. On the other hand, there is a similarity in the uses of some agricultural tools from plants between southern Yemen, Ethiopia and Pakistan [18, 19].

Until today, local people in southern Yemen still considered wild plants as main natural source for making tools of agriculture, handicrafts, building and other domestic purposes. This traditional knowledge in southern Yemen is still passed down orally from one generation to the next and it has been not documented before, except some uses of wooden agriculture tools and use of cosmetic in Hadhramout in southern Yemen [11, 16].

However, this traditional knowledge faces more danger, due to the increase in non-natural products and tools through the industry and the import from the outside, the reliance on local plants decreased in traditional uses, especially in the use of in agriculture tools, construction wood, ropes and dyes. Moreover, migration, urbanization and globalization affected the interdependence of young generations with environment, nature and plants, and this led to a decrease in their level of traditional knowledge. Generally, in Yemen and Arabian Peninsula countries, the ethnobotany of this traditional knowledge is faster to be lost in the face of urbanization in the modern era due to the uses of industrial materials [20].

However, people in most parts of the country still depend on nature more than relying on tools and continuous materials due to war and poverty, so Yemen still gives a picture of the interaction between social-economic conditions and daily needs from multiple local and external sources and from natural and industrial sources [21].

Therefore, it was necessary to document these traditional uses of traditional tools from local wild plant in southern Yemen, which took place nearly twenty years ago, to preserve the historical record of the ancient traditional uses of plants in human life in southern Yemen. These include domestic uses to improve the level of the daily life of the local people, making other important traditional tools of useful handicraft, agricultural and building tools.

## Materials and methods

### Study area

**Fig. 1:**
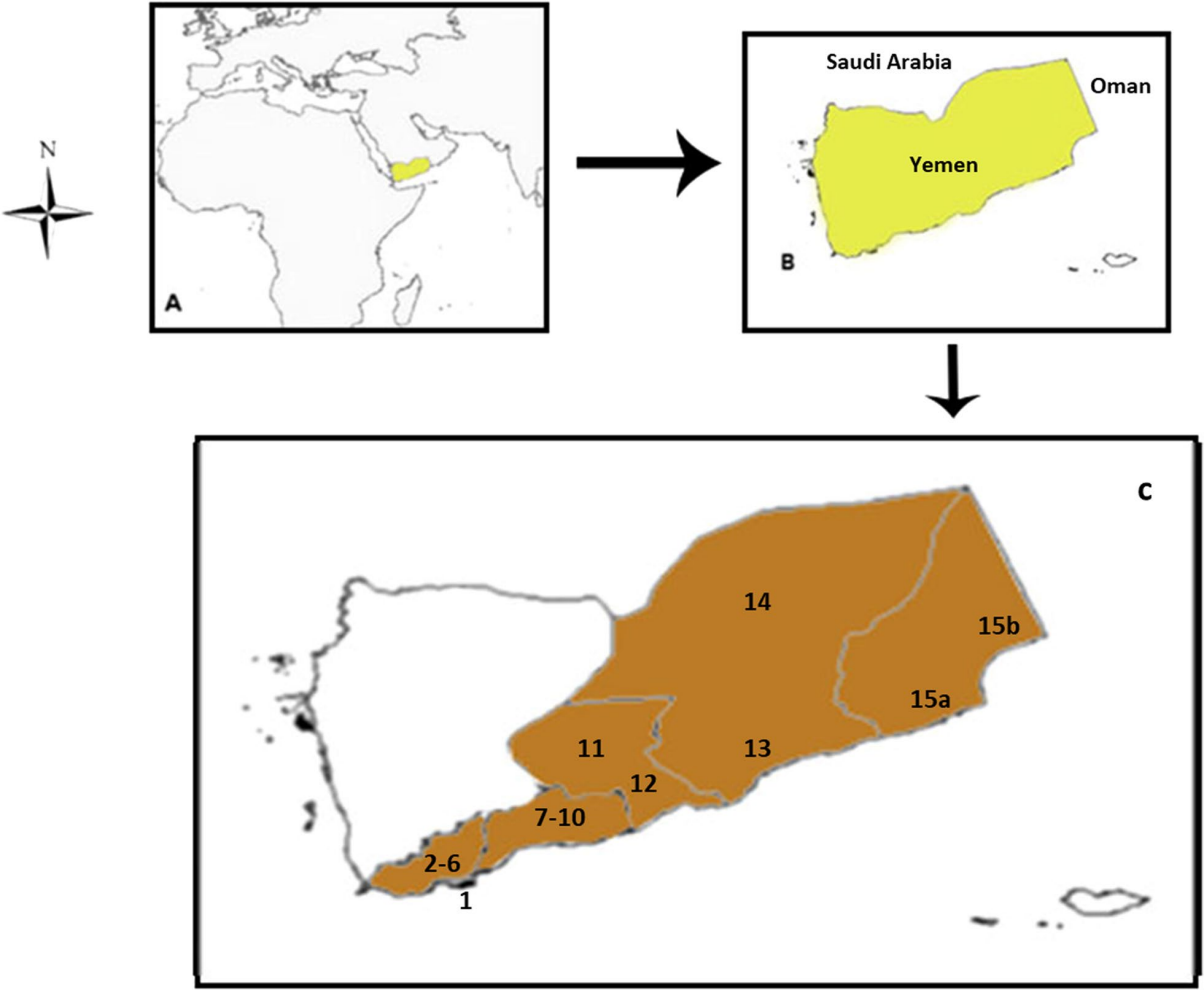
The locality of the study area. **A** Map of Yemen in the world, **B** map of Yemen, **C** map of southern Yemen in brown color, (1) Aden (Alhisawa), (2a) Lahj Al-Hawtah, (2b), Lahj (Tor Albaha) (3) Radfan, (4) Halmeen, (5) Al-Dhalee, (6a) Yafee (Sarar), (6b) Yafee (Rused), (7a) Abyan (Zingibar), (7b) Abyan (Batis), (8) Mukeiras, (9a) Lawdar, (9b) Dathina, (10) Al-Wadhee, (11) Shabwa Ataq, (12) Shabwa (Haban), (13) Hadhramaut (Syoon, Tream), (14) Hadhramout (Mukalaa), (15a) Al-Mahrah (Qeedhah) and (15b) Al-Mahrah (Haoof)

Southern Yemen is located in south of Arabian Peninsula, between Oman to east, Saudi Arabia and north Yemen to north, and Arabian sea to south (Fig. 1). Southern Yemen constitutes the largest part of the south of the Arabian Peninsula, and its phylogenetic phyto-diversity is considered part of the flora of the Arabian Peninsula, in addition, there is a similarity with the flora of the Horn of Africa. This geographical diversity led to a wide variety of wild plants such as trees, herbs and grasses. Most common wide diverse wild plants species belong to Fabaceae *(Acacia*), Euphorbiacae and Apocynaceae*.* In whole Yemen, the Apocynaceae family occupies 131 species, of which 46 are endemic, Fabaceae (205 species) and Euphorbiaceae (106 species) [22].

The agro-ecology of the study area (southern Yemen) has diverse  terrains: a coastal strip extending 1400 km, middle altitude, high altitude, valleys (wadies) and desert (Rub al-Khali) in the north-east of the country [1, 23, 24]. In Yemen in general, deserts constitute more than half of the total land area (57%). The remaining area (40%) constitutes plains, pastures, and scattered small forests, and a small percentage (3%) is arable land that produces rich crops for the country [25], while in southern Yemen, the arable area is only 0.7% of the total land area. This percentage decreases due to lack of water and lack of rain [26].

South Yemen is characterized by semi-tropical weather, often semi-desert. It is blown by monsoon winds that are generally dry. Rainfall falls in winter from October to April, but in a low and irregular manner. The weather is similar throughout the Arabian Peninsula. Rainfall usually occurs in the winter, between December and April. In summer, the rain is less. However, the rain falls in southern Yemen once a year and for a short period. It stops for many years in some areas. The average temperature in southern Yemen in general is estimated at 27 °C. In winter, the average temperature is 23–25°C. In summer, the highest temperature reaches between 33 and 44 °C [23]. There is no significant difference between southern Yemen and neighboring countries such as Oman, Saudi Arabia and the countries of the Arabian Peninsula in terms of weather, climate and terrain. This is reflected in the similarity of flora in the countries of the Arabian Peninsula neighboring Yemen. In the center of southern Yemen, there are agricultural lands that are mostly grown major crops such as wheat, sorghum (fodder and grains), millet, maize, sesame, cotton tomato, potato, dates and bananas, where the cultivated areas depend on rare flash floods, and ground water and the rest are rainfed. [26, 27]. In addition, each region in the south has ecological characteristics suitable for growing certain productive plants, for example, the cultivation of cotton in Abyan Governorate, the cultivation of henna, palm trees and tobacco in Hadhramout Governorate. In general, grains still constitute seventy percent of crops, but qat cultivation has taken 11% of agricultural production, especially in most regions of northern Yemen [25], while only in two regions in southern Yemen in Al-Dhalee and Yafee. The cultivation of qat poses a devastating threat to crop cultivation and the economy in the country. In the past thirty years, agriculture has faced a serious threat from water shortages due to increasing population growth, the lack of sustainable rains and the lack of a stable economy due to war, and on top of that, water is consumed in the cultivation of qat (*Catha edulis*) [28]. Therefore, Yemen is classified as a poor country [29]. In the last three decades, mass migration from rural to urban areas has escalated significantly due to the escalation in population growth, random urban expansion on private agricultural lands, lack of agricultural production and food insecurity due to wars. All of this led to significant neglect or abandonment of agricultural lands in many areas of the country. In addition, there is permanent migration from Yemen to rich countries such as the countries of the Arabian Gulf, the Arabian Peninsula and the Western world.

### Data collection

Ethnobotanical data were collected during the periods 2000–2005, using open-structured interviews with direct observation focusing on traditional wild plants for domestically traditional uses. The local informants interviewed were 1020 (1851 men, 169 women) (Table 1). Their ages ranged from 30 to 80 years. Most of the interviewees were more than 60 years old. The informants were farmers, shepherds, housewives, student and men working in beekeeping. Most of them are elderly. All informants were spoke Arabic language. Interviews were carried out in the field, where the informants were asked to find the fresh plant specimens on the study are or by the vernacular name of the plant. Voucher specimens were kept in the Al-Fatimi's Herbarium, Aden, Yemen.

**Table 1:** Details of the visited locations and the informants involved; all informants are Arab and Muslim speak Arabic language

N	Location	Area	Altitude meter (m)	GPS coordinates	Total inhabitants	Range of age	F	M	Total informants
1	Aden (Alhisawa)	P	2	12° 49′ 52″ N; 44° 56′ 23″ E	1,053,455	30–70	8	16	24
2a	Lahj (Al-Hawtah)	H	134	13° 03′ 9″ N; 44° 52′ 44″ E	36,186	45–80	10	40	50
2b	Lahj (Tor Albaha)	H	442	13° 00′ 30″ N; 44° 27′ 12″ E	68,726	50–70	5	16	21
3	Radfan (Al-Habeleen)	MO	621	13° 30′ 59″ N; 44° 51′ 50″ E	62,698	55–80	6	20	26
4	Halmeen	H	938	13° 38′ 31″ N; 44° 51′ 11″ E	40,678	59–80	7	36	43
5	Al-Dhalee	H	1507	13° 42′ 18″ N; 44° 43′ 41″ E	135,886	50–70	9	66	75
6a	Yafee (Rused)	MO	1690	13° 42′ 31″ N; 45° 17′ 55″ E	75,501	59–70	12	20	32
6b	Yafee (Sarar)	MO	1686	13° 38′ 43″ N; 45° 20′ 43″ E	21,064	59–70	2	28	30
7a	Abyan (Zingibar)	H	22	13° 07′ 29″ N; 45° 21′ 55″ E	41,176	65–80	10	34	44
7b	Abyan (Batis)	H	154	13° 07′ 29″ N; 45° 21′ 55″ E		65–80	4	34	38
8	Mukeiras	MO	2172	13° 56′ 09″ N; 45° 40′ 49″ E	50,220	64–85	8	45	53
9a	Lawdar	H	975	13° 52′ 43″ N; 45° 52′ 9.4″ E	122,909	55–80	25	67	92
9b	Dathina (Modyah)	H	837	13° 55′ 38″ N; 46° 04′ 45″ E	48,933	55–85	18	66	84
10	Al-Wadhee	H	821	13° 42′ 48″ N; 46° 00′ 41″ E	32,652	55–70	9	59	68
11	Shabwa Ataq	H	1160	14° 31′ 35″ N; 46° 51′ 09″ E	56,001	65–80	6	31	37
12	Shabwa (Haban)	H	961	14° 20′ 35″ N; 47° 04′ 0.4″ E	42,684	50–75	4	27	31
13	Hadhramout (Syoon)	H	1511	14° 54′ 46″ N; 48° 11′ 49″ E	161,538	50–80	5	76	81
14	Hadhramout (Mukalaa),	P	43	14° 32′ 54″ N; 49° 08′ 0.8″ E	314,942	30–70	16	89	105
15a	Mahrah (Qeedhah)	P	16	16° 12′ 27″ N; 52° 11′ 43″ E	57,120	59–70	3	42	45
15b	Mahrah (Haoof)	MO	1072	16° 41′ 10″ N; 52° 48′ 15″ E	9,663	55–80	2	39	41

Altitude (P = plain, H = hill, and MO = mountain); GPS coordinates; total number of inhabitants (at the time the interviews were carried on); and gender (M: male; F: female), distribution, range of age of the interviewees, and total number of interviewees. The numbers in the first column indicate the numeration consistent with the ones reported in the map given in Fig. 1

Twenty rural and urban villages and cities were selected to carry out the field investigations (Table 1). The identification and nomenclature of the listed plants were based on southern Yemen flora [30–32]. In the botanical list of Table 2 are reported for each plant: scientific name, voucher herbarium number, family, vernacular name, parts used, way of use, folk domestic uses and localities where the use was collected, number of localities for each use. For each species the vernacular name, the way it is used and the locations of traditional use are given. Scientific names of studied plant species and the conservation status of the reported plant species were confirmed by using the references of World Flora Online (http://www.worldfloraonline.org) (WFO, 2021), and Global Biodiversity Information Facility (GBIF) (https://www.gbif.org/).

**Table 2:** Traditional general uses of wild plant species in southern Yemen

Botanical data of the plants: Scientific name/Family/Growth form/Voucher specimen no	Local name of plant species (locality)	Used parts	Traditional uses (citation number per use category)	No Cit.
*Aerva javanica* (Burm.fil.) Juss. ex Schult./Amaranthaceae/Herb MSY 015	Ra را (2–15)	Fruit	*Handicraft*: Fiber: Pillows making: Fruits (diaspores) for fill sleeping pillows and saddle of donkey (100)	100
*Aerva lanata* (L.) Juss./(*Ouret lanata* (L.) Kuntze) Amaranthaceae/Herb MSY 016	Ra را (5,13)	Fruit	*Handicraft*: Fiber: Pillows making: Fruits (diaspores) for fill sleeping pillows and saddle of donkey (44)	44
*Aloe inermis* Forssk./Asphodelaceae/Shrub MSY 012	Saber صَبِر (6–10, 13–15)	Leaf Juice	*Economic value*: *Aloe* sap mixes with the black dye left by the smoke from burning wood. The mixture is used as ink for writing and is not erased. The juice is mixed with black smoke to product pens ink. Mix the liquid with curcuma as base (35)	68
		Dried leaf	*Health aid*: Insect repellant: Burns its fumigant used as insect repellant. The dried leaves burn as fumigant during childbirth (10)	
		Flower	*Beekeeping* Bee forage: Flowers are important nutritional source for honeybees (18)	
		Entire plant	*Ornamentals*: Decorative plants on graves (5)	
*Aloe vacillans* Forssk Asphodelaceae/Shrub MSY 013	Saqel صَقِل (6,8,9)	Leaf Juice	*Economic value*: Ink product: *Aloe* sap mixed with the black dye left by the smoke from burning wood. The mixture is used as ink for writing and is not erased. The juice is mixed with black smoke and curcuma to product pens ink (15)	41
		Leaf	*Health aid:* Insect repellant: The dried leaves urn as fumigant during childbirth (21)	
		Entire plant	*Ornamentals:* Decorative plants on graves (5)	
*Aloidendron sabaeum* (Schweinf.) Boatwr. & J.C.Manning**/ (= *Aloe sabaea* Schweinf.)/ Asphodelaceae/Shrub MSY 014	Qubub قُبُب (2–15)	Stem	*Construction timber*: The stems are dried, collected and described next to each other, tied together with sticks, and doors are made of them (45)	45
*Amaranthus graecizans* L./ Amaranthaceae/Herb MSY 049	Dhadah, ضدح Ladah لدح (8,9,10)	Entire plant	*Animal husbandry: Livestock fodder*: delicious forage for all livestock The milk of goats eating this herb is rich and sweet with distinctive and high nutritional values (56)	56
*Anisotes trisulcus* (Forssk.) Nees/Acanthaceae/Shrub MSY 006	Mudhadh مضاض (5,7–10, 11,13)	Stem	*Construction timber*: Stems are used for making house ceiling (as Roof thatching with timber of other species) (100)	173
		Flower	*Beekeeping Bee forage:* Flowers are important nutritional source for honeybees (70)	
		Dead wood	*Fuel wood*: Good firewood (3)	
*Artemisia abyssinica* Sch.Bip. ex A. Rich *Artemisia abyssinica* Sch.Bip. ex Oliv. & Hiern /Asteraceae/Herb MSY 026	Schuqur-Abiadh شقر ابيض (6,8)	Entire plant	*Health aid:* Pre*servative agent:* Herb fresh raw cut and put in rooms and between document papers, to preserve against insect attack (5) *Health aid: Insect repellant:* and it is placed between the clothes to repel insects (15)	70
			*Ornamentals:* Houseplant ornamental (50)	
*Balanites aegyptiaca* (L.) Delile/ Zygophyllaceae/Tree MSY 029	Surr صُر (8–13)	Wood	*Agriculture tools*: Wood is used for making wooden plow (plough) "*mehrath*" *Construction timber*: for house door and ceiling (78)	78
*Blepharis ciliaris* (L.) B.L. Burtt/ Acanthaceae/Herb MSY 008	Showak شوك (2–11)	Leaf	*Health aid*: *Insect repellant:* The squeezed liquid of the plant fresh leaves is sprayed at house to kill fleas (6)	26
			*Animal husbandry*: *livestock fodder*: for camel (20)	
*Boscia arabica* Pestal./ Capparaceae/Tree MSY 046	Sarh سرح (8–14)	Wood	*Construction timber*: timber is used for making house door and ceiling (65)	65
*Breonadia salicina* (Vahl) Hepper & J.R.I.Wood.*/* Rubiaceae/Tree MSY 072	Dhrah ضرح (5–6)	Bark	*Handicraft*: *Defense tools*: The bark is used to make a wick for setting fire to old guns (5)	5
*Calotropis procera* (Aiton) Aiton fil./ Apocynaceae /Shrub MSY 011	Ushur عُشر (6–15)	Leaf, latex	*Economic value*: *Tanning agent:* Leaves and latex are used to remove the hair of animal as leather tanning (50)	72
		Stem	*Agriculture tools*: hollowing out of a branch stick (*methrah*) connected to the wooden plow to distribute the seeds during tillage (12)	
			*Handicraft*: *Defense tools*: In the past, they used to mix the ashes of sticks with sulfur and salt to make gunpowder (5)	
		Bark	*Handicraft: Fiber*: cordage, rope "*habl*" (5)	
*Commiphora gileadensis* (L.) C.Chr./Burseraceae/Shrub MSY 017	Besham بشام (6–13)	Stem, wood	*Agriculture tools*: Trunk used as a wooden grain measure. Wooden yoke, wooden plow (plough) (10)	15
		Stem, wood	*Fuel wood*: Dried stem firewood (5)	
*Commiphora kataf* (Forssk.) Engl./Burseraceae/Shrub MSY 018	Qataf قطف (8,9)	Wood, stem	*Agriculture tools*: Trunk used as a wooden grain measure. Wooden plow (plough) (*mehrath*).. Wooden yoke (21)	32
			*Fuel wood*: Dried stem firewood *Handicraft: Cook/Kitchen tools:* Wooden pestle *(minhaz)* used to crush vegetables (11)	
*Corchorus depressus* (L.) Stocks Malvaceae /Herb MSY 040	Legen لِجِن (7–10)	Leaf	*Health aid*: *Cleansing:* Leaves powdered mixed in water smear hair for some timed and wash of hair to clean (33)	33
*Corchorus trilocularis* L./ Malvaceae/Herb MSY 041	Legen لِجِن (7–10)	Leaf	*Health aid*: *Cleansing:* Leaves powdered mixed in water smear hair for some timed and wash of hair to clean (33)	33
*Cordia quercifolia* Klotsch./ Cordiaceae/Tree MSY 019	Sahel سحل (6–10)	Stem. wood	*Agriculture tools:* Wooden plow (plough) (*mehrath*). A wooden yoke (*heig*) (11)	99
		Stem	*Handicraft: Defense tools:* a stick used as defense tool called (*assa, mohwer*) (10)	
		Stem	*Handicraft: Cook/Kitchen tools:* A stick used as manual mixer for mixing water and flour during cooking it called (*medhrar*). Wooden bowl (mortars) and pestles (8)	
		Stem	*Agriculture tools:* Wooden plow (plough) (*mehrath*) (20)	
		Wood	*Construction timber:* Wood for the ceiling (50)	
*Cordia sinensis* Lam/Cordiaceae/Tree MSY 020	Sahel سحل (8–11)	Stem	*Agriculture tools:* Wooden plow (plough) (*mehrath*) (10)	50
		Wood	*Construction timber:* Wood for the ceiling (40)	
*Cynanchum vanlessenii* (Lavranos) Goyder*/Apocynaceae/Shrub *(*= *Sarcostemma vanlessenii* Lavranos) MSY 050	Qalab غلب (8–10)	Stem	*Health aid: Insect repellant:* Stems repels insects (11)	11
*Cynanchum viminale* subsp. *stipitaceum* (Forssk.) Meve & Liede/ (= *Sarcostemma viminale* (L.) R.Br. subsp. *stipitaceum* (Forssk.) Meve & Liede)/Apocynaceae/Shrub MSY 051	Alab, Leb الب، لب (8–10)	Stem	*Health aid:* *Insect repellant:* Stems are burn to repel insects (15) *Preservative agent:* Dried piece of stem, burn and fumigant let in the room after birth and for cloths of new births (8)	23
*Cynanchum viminale* subsp. *viminale* /Apocynaceae/Shrub (*Sarcostemma viminale* (L.) R.Br.) MSY 052	Alab, الب Leb لب (8–10)	Flower	*Beekeeping bee forage:* The flowers are important nutritional source for bee to produce honey (45)	50
		Stem	*Health aid: Insect repellant:* Dried stems dried pieces' burn, fumigant let in the room against insect attack, after birth (5)	
*Cyphostemma digitatum* (Forssk.) Desc. /Vitaceae/Climber MSY 042	Halqah حلقه (13,14)	Leaf	*Economic value: Tanning agent:* Leaves for tanning of animal skins for leather making (23)	23
*Dracaena forskaliana* (Schult. & Schult.f.) Byng & Christenh./Asparagaceae/Herb (= *Sansevieria forskaliana* (Schult. & Schult.f.) Hepper & J.R.I.Wood)/ MSY 021	Khraq, خرق Gazab جزاب (6)	Leaf	*Handicraft: Fibers:* Leaf put in water for some days to soften; it used for thick rope "*habl*" (12)	12
*Dracaena hanningtonii* Baker/Asparagaceae/Herb (= *Sansevieria ehrenbergii* Schweinf. ex Baker) MSY 022	Seni سني (6–10)	Leaf	*Handicraft: (Fibers)*: Leaves are used for making fibers, thin ropes for weaving rope and sling-like (*walaf*). The fibers were loosened from the leaves of the plant and wrapped and twisted on the thigh; this process is called "*sehieg*" (100)	100
*Dracaena serrulata* Baker**/Asparagaceae/Tree MSY 023	Arab, عراب Arabah, عرابه Khaws, خوص Azaf عزف (5,6,13,14, 15)	Flower	*Beekeeping*: *Bee forage:* flower for Bee honey (5)	22
		Stem	*Beekeeping*: making hive for bee (5)	
		Leaf	*Handicraft:* Fibers for rope and cord "*habl*" making. Baskets and natural mat from leaves (12)	
*Ehretia cymosa* Thonn./Ehretiaceae/Shrub MSY 002	Warf ورف (5)	Stem	*Agriculture tools*: It makes a stick from the stems of the plant that is used as a beater to separate the wheat grains from the wheat spike. The stick is called locally (*malbag*) (6)	6
*Euphorbia cactus* Ehrenb. ex. Boiss./Euphorbiaceae/Shrub MSY 030	Qassas, قصاص Karath ^كراث^ (2–14)	Flower	*Beekeeping*: Bee forage: flower nectar gives a pungent taste of the honey (56)	56
*Euphorbia granulata* Forssk.*/* Euphorbiaceae /Herb MSY 031	Mehlabieh, ملبنه Melebeneh مليبنه (2–14)	Entire plant	*Animal husbandry*: *Milk production:* Herb increases milk of livestock (10)	10
*Euphorbia inaequilatera* Sond Euphorbiaceae/Herb MSY 032	Melebenh مليبنه (6,8)	Entire plant	*Animal husbandry: Milk production:* Herb increases milk of livestock, intensively grazed by livestock (24)	24
*Euphorbia radfanensis* Al-Fatimi & N.Kilian Euphorbiaceae/Herb MSY 033	Qasas قصاص (5)	Entire plant	*Animal husbandry*: *livestock fodder*: dried heb grazed by camels (30)	40
		Flower	*Beekeeping*: Bee forage: flower nectar gives a pungent taste of the honey (10)	
*Euryops arabicus* Steud. ex Jaub. & Spach Asteraceae/Herb MSY 073	Shagart alkns شجرة الكنس (5,11,14)	Entire plant	*Handicraft:* House cleaning tools: Brooms (4)	4
*Fagonia indica* Burm.fil./ Zygophyllaceae/ Herb MSY 010	Hell حل (8–10,14,15)	Dried stem	*Fuel wood:* Very flammable, so it is used to help lightwood as firelighter (44)	44
*Ficus cordata* Thunb./ Moraceae/Tree MSY 058	Athab, اثب Theb ثب (2–15)	Bark	*Animal husbandry:* *Livestock fodder:* livestock graze dead bark (4) *Milk production*: Barks powder in milk to assist to become clot (4)	98
			*Handicraft: Defense tools:* The bark is used to make a wick for setting fire to ancient guns (8)	
			*Handicraft: Fibers:* for ropes making (5)	
		Stem, Wood	*Health aid: Preservative agent:* A cavity is drilled in the stem of the plant to be used as a container to store water for livestock to drink from. It is said that the plant stem prevents the attack of insects and against microbial contamination (6)	
			*Construction timber:* House roof timber, windows (11)	
			*Agriculture tools:* Wooden tillage; wooden grain measure. Wooden trough for water drinking of livestock. Fencing: dried stems are cut and used to protect the coffee, corn and millet plants (52)	
			*Beekeeping:* wood and stem for making hive for bee (8)	
*Ficus palmata* Forssk./Moraceae/ Tree MSY 059	Balas بلس (6,8)	Latex	*Animal husbandry: Milk production: Flavoring agent:* Two drops of the fruit latex added to the milk (*labah*) turns into buttermilk (*dahen*) (8)	16
			*Construction timber:* Doors and windows (8)	
*Ficus sycomorus* L./ Moraceae/Tree MSY 060	Saqam سقم (2–8)	Stem, wood	*Beekeeping*: Wooden beehive (5)	17
			*Construction timber:* Its wood is used in making doors (*saddah*), and locks and stairs (8)	
			*Agriculture tools:* Wooden tillage tools; wooden grain measure (4)	
*Ficus vasta* Forssk./ Moraceae/Tree MSY 061	Tauluq تولق (2–15)	Wood, stem (trunk)	*Construction timber:* Wood for the manufacture of locks and keys for wooden doors (*saddah*) (27)	55
			*Beekeeping*: Wooden beehive (15)	
		Bark	*Handicraft: Fiber:* for ropes making (3)	
		Entire tree	*Health aid: Shade tree:* Important trees as shades for people and animals (10)	
*Grewia tembensis* Fresen.*/* Malvaceae/Shrub MSY 053	Schohudt شوحط (5, 8–10)	Stem	*Agriculture tools:* The bark is removed from stem that used as stick to hit Spike-Weizen to separate the grains (*moher*). Stem can be used wooden handle for shovel (*magrafah*) (15)	40
			*Handicraft: Cook /Kitchen tools*: A thick stick used as manual mixer for mixing water and flour during cooking it called (*medhrar*; *mahwasch*) (10)	
			*Fuel wood:* Firewood (5)	
			*Health aid:* Wooden walking stick (10)	
*Grewia tenax* (Forssk.) Fiori/ Malvaceae/Shrub MSY 054	Schohudt, شوحط Hanqlis حنقلس (2–10)	Stem	*Agriculture tools:* Stems are made of sticks to hit the Spike-Weizen to separate the grains. Wooden handle for the axe (*fas*) (15)	30
			*Fuel wood:* Firewood (5)	
			*Health aid:* Wooden walking stick (10)	
*Grewia trichocarpa* Hochst. ex A.Rich. /Malvaceae/Shrub MSY 055	Neshem نشم (6–10)	Stem	*Agriculture tools:* Stems are made of sticks to hit Spike-Weizen to separate the grains (*moher*) (15)	15
		Wood	*Fuel wood:* good firewood (5)	5
*Hyparrhenia hirta* (L.) Stapf/ Poaceae/Grass MSY 047	Namas نمص (2–6)	Inner bark	*Handicraft: Fibers:* The delicate inner barks of stems are a binding material in the manufacture of containers (13)	13
*Hyphaene thebaica* (L.) Mart./ Arecaceae/Tree MSY 009	Bahesh بهش Tari طاري (1)	Stem, Liquid exudate, Fruit	*Economic value: Refreshing drink:* Liquid exudate from incision of stem used as drink. It is used as a refreshing, relaxing and cheerful drink. This drink locally called “*tari*” (15); it is called " local vinegar" *Economic value: Refreshing drink:* Liquid of fruit used as refreshing drink (5)	26
		Wood	*Economic value:* Wood used for boat manufacturing (3)	
		Leaf	*Handicraft: Fibers:* Leaves are used to make rope *"habl*", broom "*maknasah*", basket "*salah*", mat *"hassir*", hat "*qubaah"* (3)	
*Indigastrum costatum* (Guill. & Perr.) Schrire/Fabaceae /Shrub MSY 034	Hewer حيور (6–8)	Leaf	*Economic value: Dyestuff:* Leaves powder mixture with water and use as black dyestuff for hair (11)	11
*Indigofera arabica* Jaub. & Spach/Fabaceae/Shrub MSY 035	Shubrum شبرم (7–14)	Entire plant (small shrub)	*Health aid: Preservative agent*: Fresh raw plant, rub the wooden pots. Parts burn in use its fumigant against microbial contamination, water preservative (6)	6
*Indigofera coerulea* Roxb./Fabaceae /Shrub /MSY 036	Hewer حوير (7–14)	Entire plant	*Economic value: Dyestuff:* Entire plant cut mixed in water boiling, mixed with cloths to give dark blue color (46)	46
*Indigofera tinctoria* L./Fabaceae/ Shrub/MSY 037	Hiwer, حيور Katem, كتم Nil نيل (6–10)	Leaves	*Economic value: Dyestuff*: Leaves powder mix with water and use as black dyestuff for hair and cloths (80)	80
*Kleinia odora* (Forssk.) DC./ Asteraceae/Shrub MSY 044	Khidhaan, قيطان Khulaan, خولعان Audher عضور (6–10)	Stem juice	*Health aid: Preservative agent:* Dried juice burns fumigant used as incense. It is used as incense after childbirth (12)	12
*Lycium shawii* Roem. & Schult./ Solanaceae/Shrub MSY 048	Awseg عوسج (2–15)	Stems	*Agriculture tools:* It makes a stick from the stems of the plant that is used as a beater to separate the wheat grains from the wheat ears of wheat. It is called the stick locally (*malbag*) (78)	78
*Nannorrhops ritchieana* (Griff.) Aitch Arecaceae /Tree MSY 005	Tari طاري (14, 15)	Leaf	*Handicraft: Fiber:* Rope is made from leaf fiber. Mat, Hut (30)	30
*Ocimum forsskalii* Benth Lamiaceae/Herb MSY 001	Huqiqab, حقيقاب Dhomaran ضومران (7–10)	Entire plant	*Ornamental:* The plant is used to spread the pleasant smell in the rooms and places. The plant is also planted on graves (46)	46
*Olea europaea* subsp*. cuspidata* (Wall. & G.Don) Cif./Oleaceae /Tree MSY 043	Attem عتم (6,8)	Wood, stem	*Construction timber:* Wood and stem are used for building houses and making house ceiling (15)	39
			*Handicraft: Cook /Kitchen tools:* for making a long wood to move cooked Mel with water, this wood called (*medhrar*) (14)	
			*Animal husbandry:* Shepherd's stick (10)	
*Pulicaria undulata* (L.) C.A. Mey./Asteraceae/Herb MSY 003	Gethgath, جثجات Gifgaf جفجاف (2–10)	Entire plant	*Handicraft: House cleaning tools:* Dried herb is used for making as broom for house cleaning; and after cleaning, room air has aromatic smell obtained from the aromatic plant (57)	82
			*Agriculture tools:* To purify grains to remove impurities by sweeping (25)	
*Reichardia tingitana* (L.) Roth/ Asteraceae/Herb MSY 024	Melebena ملبنه (6–10)	Leaf	*Animal husbandry Milk production:* Increase milk production by livestock (43)	43
*Salvadora persica* L./ Salvadoraceae/Shrub MSY 045	Rak راك (2–15)	Leaf	*Animal husbandry Milk production:* nice smell for milk of livestock when eating (11)	11
*Schoenoxiphium sparteum* (Wahlenb.) C.B.Clarke/ Cyperaceae/Grass MSY 056	Aazaf عزف (6,8)	Leaf	*Handicraft: Fiber:* Rope is made from leaf fiber. Mat and broom are made from leaf (5)	5
*Searsia retinorrhoea* (Steud. ex Oliv.) Moffett/Anacardiaceae/Shrub MSY 007	Taleb طلب (5–8)	Stem	*Agriculture tools:* Wooden stick is made as an ax handle (8)	8
*Senegalia asak* (Forssk.) Kyal. & Boatwr./ (= *Acacia asak* (Forssk.) Willd.)/ Fabaceae/Tree MSY 062	Asaq عسق (2–10)	Flower	*Beekeeping*: *Bee forage: Acacia* flowers are important nutritious forage for honeybees (50)	77
		Stem	*Handicraft: Cook /Kitchen tools:* A solid stick without bark used by hand to mix flour with water during cooking. The food made by this method is called locally (*zad*) and the stick mixer is called (*medhrar*) Agriculture tools: Used stick to separate the grains from spike *(malbag*). Wooden plow (plough) by ox (10)	
		Stem, Wood	*Fuel wood:* best firewood (5)	
		Leaf	*Animal husbandry*: *Livestock fodder*: common and good forage mainly for goats and camels (12)	
*Senegalia hamulosa* (Benth.) Boatwr./ (= *Acacia hamulosa* Benth.)/Fabaceae/Tree MSY 063	Qatad, قَتاد Qutad قُتاد (2–4,8,11,14)	Wood	*Agriculture tools:* Wooden plow (plough) (*mehrath*) by ox (21)	56
			*Handicraft: Cook /Kitchen tools:* wooden cups (13)	
		Stem, gum	*Economic value:* the dried liquid exudate of the stem is high valuable Arabic gum (10)	
		Leaf	*Animal husbandry*: *Livestock fodder*: common and good forage mainly for goats and camels (12)	
*Senegalia mellifera* (Benth.) Seigler & Ebinger/ (= *Acacia mellifera* (M.Vahl) Benth.)/ Fabaceae /Tree MSY 064	Dhubian, ضبيان Dhabah ضبه (1,5,7–10,14)	Flower	*Beekeeping*: Bee forage: Flowers are important nutritional source for bee to produce the honey with high quality (55)	97
		Bark	*Health aids*: Flavoring and antiseptic agent: Barks burn and use antiseptic fumigant in the pot to get good taste and smell of food (6)	
		Stem, wood	*Agriculture tools:* Wooden plow (plough) (*mehrath*), pulled by oxen (*dhamed*) (11)	
			*Fuel wood:* firewood (13)	
		Leaf	*Animal husbandry*: *Livestock fodder*: common and good forage mainly for goats and camels (12)	
*Solanum lasiocarpum* Dunal (= *Solanum indicum* L.)/ Solanaceae/Herb MSY 025	Qumqam قمقام (7–10)	Fruit, Berries	*Animal husbandry: Milk production*: Raw seeds are mixed with milk of goats or sheep to become clot with delicious taste, after remove of seeds (63)	63
*Tamarix aphylla* (L.) Karst./ Tamaricaceae/Tree MSY 038	Waaer وعر Duaar دعر (6–15)	Wood	*Construction timber*: Dried powdered stems and leaves mixed with water and soil and use for house ceiling (17)	58
			*Economic value:* it makes good charcoal (26)	
		Wood, stem	Fuel wood: good firewood (4)	
		Entire plant	*Agriculture;* Fencing*:* The plant culitivated to repel wind and sand (11)	
*Tamarix nilotica* (Ehrenb.) Bunge/Tamaricaceae/Tree MSY 039	Athal, أثل Tarfa طرفه (2–15)	Wood, Stem, Leaf	*Construction timber:* Dried powdered stems and leaves mixed with water and soil and use for house ceiling (47)	98
		Wood	*Fuel wood:* good firewood (4)	
		Wood, stem	*Handicraft:* Cook /Kitchen tools: Wood for making a wooden cup (10)	
			*Economic value*: it makes good charcoal (26)	
		Entire plant	*Agriculture* Fencing*:* The plant cultivated to repel wind and sand (11)	
*Tarchonanthus camphoratus* L./Asteraceae/Shrub MSY 004	Qummur قُمُر (3–14)	Leaf	*Health aid:* Cleansing: Leaf powder mix with water for body wash (16)	58
		Fruit	*Handicraft:* Fiber: fruits for making pillows to use for sleep (42)	
*Typha domingensis* Pers./ Typhaceae/Herb MSY 057	Alal علعل (5, 15)	Leaf	*Handicraft:* Fiber: for ropes making (24)	24
*Vachellia etbaica* (Schweinf.) Kyal. & Boatwr./ (= *Acacia etbaica* Schweinf.) / Fabaceae/Tree MSY 065	Qaradh قرض (4,5)	Stem, wood	*Handicraft: Cook/Kitchen tools*: wooden cup, wooden bowl (50)	87
			*Agriculture tools:* wooden stems used for poles on wells to draw water in buckets (13)	
			*Economic value:* wood makes good charcoal (12)	
		Leaf	*Animal husbandry*: *Livestock fodder*: common and good forage mainly for goats and camels (12)	
*Vachellia flava* (Forssk.) Kyal. & Boatwr./ (= *Acacia ehrenbergiana* Hayne)/Fabaceae/Tree MSY 066	Dhibian ضبيان (1–15)	Stem, wood	*Fuel wood:* important firewood (5)	56
			*Economic value:* it makes good charcoal (15)	
		Leaf	*Animal husbandry*: *Livestock fodder*: common forage mainly for goats and camels (13)	
		Flower	*Beekeeping: Bee forage*: bee produced valuable honey by eating these flowers (23)	
*Vachellia gerrardii* (Benth.) P.J.H.Hurter/ (= *Acacia gerrardii* Benth.)/ Fabaceae/Tree MSY 067	Taleh طلح (5,6,8)	Leaf, fruit	*Animal husbandry: Livestock fodder:* good forage mainly for goats and camels (20)	98
		Leaf	*Economic value: Tanning agent:* Leaf used as tanning agent for making animal leather (12)	
		Stem/ wood	*Construction timber:* Wood is solid and used to make the roofs, doors and windows (45)	
			*Handicraft: Cook/Kitchen tools*: A solid stick without bark used by hand as manual mixer for mixing water and flour during cooking it called (*medhrar*; *mahwasch*) (10)	
			*Fuel wood:* high quality of firewood (5)	
		Flower, Stem (trunk)	*Beekeeping*: *Bee forage:* Flower is good forage for bee and stem for making hives (wooden beehive) (6)	
*Vachellia nilotica* (L.) P.J.H.Hurter & Mabb*./* (= *Acacia nilotica* (L.) Willd. ex Delile)* /* Fabaceae /Tree MSY 068	Qaradh قرض (1–15)	Leaf	*Health aid: Preservative agent:* Leaves powder mixed in water as preservative agent (water called *qaral*). Leaves fumigant to clean wooden container (12)	378
			*Economic value:* Tanning agent: Leaves used as tanning agent for making animal leather preparation (100)	
		Gum	*Economic value:* the most important Arabic gum is produced by incision of the stem (50)	
		Stem, Wood	*Agriculture tools:* Stems for poles on wells to draw water in buckets. Wooden plow (plough) (*mehrath*) by oxen (*dhamed*). Handle of sickle (*masrab*) from wood for harvesting process (63)	
			*Handicraft: Cook /Kitchen tools*: wooden cups, wooden bowl (*kuaadah*) (54)	
			*Construction timber*: Stems and wood are used for making house roof (timber) (87)	
		Leaf	*Animal husbandry*: *Livestock fodder*: common and good forage mainly for goats and camels (12)	
*Vachellia seyal* (Delile) P.J.H.Hurter/ (= *Acacia seyal* Delile)/Fabaceae/Tree MSY 069	Taleh طلح (3,5,6)	Wood	*Handicraft: Cook/Kitchen tools:* Wooden pestle for crushing vegetables, onions and garlic (50)	58
		Leaf	*Animal husbandry*: *Livestock fodder*: common and good forage mainly for camels (8)	
*Vachellia tortilis* (Forssk.) Galasso & Banfi/ (*Acacia tortilis* (Forssk.) Hayne)/ Fabaceae /Tree MSY 070	Sumor سُمُر (2–15)	Flower	*Beekeeping*: *Bee forage:* Flowers are important source for nutritious forage for honeybees, premium honey known in southern Yemen (*asal sumur*) (*Acacia* honey) with high value for its common medicinal and nutritional uses (100)	459
		Leaf, fruit	*Animal husbandry*: *Livestock fodder*: An important and high nutritious forage for all livestock especially goat and camel to produce the best meat of livestock (100)	
		Stem	*Animal husbandry*: spiny stems dried, cut and used as fence for livestock or fields (23)	
		Stem, Wood	*Construction timber:* Stems and wood are used for making house roof (timber) (15)	
			*Economic value:* Wood provides good charcoal (100)	
			*Fuel wood:* common important source for firewood due to it its large distribution abundance in the study area (21)	
		Entire plant	*Health aid:* Shade tree: Tree provides shade for human and animal (100)	100
Vachellia yemenensis* (Boulos) Ragup., Seigler, Ebinger & Maslin/ (*Acacia yemenensis* Boulos)/ Fabaceae /Tree MSY 071	Habalah حبله (5)	Stem, Wood	*Agriculture tools:* It makes a stick from the stems of the plant that used to separate the wheat grains from the wheat spike. This stick is called locally (*malbag*) (5)	5
Ziziphus leucodermis** (Baker) O.Schwartz/ Rhamnaceae/Tree MSY 027	Habidh حيبض (14,15)	Wood, Stem	*Construction timber:* in the manufacture of main locks of door, door, windows, house ceiling (30)	40
		Leaf	*Health aid: Cleansing*: Leaves suspended in water to wash hair (10)	
*Ziziphus spina-christi* (L.) Desf./Rhamnaceae/Tree MSY 028	Elb علب (1–15)	Leaf, Bark	*Health aid: Cleansing:* Leaves suspended in water *"qasel*;" is used to wash hair and body. Barks mixed in little water as paste "*lagub*" and used as shampoo to wash and clean women hair (100)	514
		Flower	*Beekeeping*: *Bee forage:* Its flowers are the best food for bees. Flowers are important source for bee honey, which is high, valued for its medicinal and nutritional uses. The honey called local "*asal ilb* " (102)	
		Wood, Stem	*Construction timber:* in the manufacture of doors (*saddah*), windows, house ceiling, locks and keys of doors (100)	
			*Agriculture tool:* Wooden plow (plough) by two oxen using yoke. Three wooden poles used on the well to draw water in a bucket (88)	
			*Handicraft: Cook /Kitchen tools*: wooden bowl (*kuaadah*), wooden plate. A thick wooden stick (*minhaz*) for pounding the grains (12)	
			*Economic value:* Fixed oil product: Wooden oil mill: From thick trunk, a wooden machine is made to crush and squeeze the seeds to extract the oil from the seeds of sesame plant (*Sesamum indicum* L.). This traditional wooden presser for seed oil is called (*maasarah*) which is still even in the cities (30)	
		Seed	*Economic value:* Oil product: Seeds for oil production (30)	
		Entire plant	*Health aid: Shade tree:* Important trees as shades for people and animals *Agriculture tool:* Fencing*:* Trees are planted as or hedge for fields (35)	
			*Animal husbandry*: Dead thorny plant is used for livestock fences (5)	
		Leaf, fruit	*Animal husbandry*: *Livestock fodder*: common and good forage mainly for goats and camels (12)	

No Cit.: citations number. Localities: 1–15, (1) Aden (Alhisawa), (2a) Lahj Al-Hawtah, (2b), Lahj (Tor Albaha) (3) Radfan, (4) Halmeen, (5) Al-Dhalee, (6a) Yafee (Sarar), (6b) Yafee (Rused), (7a) Abyan (Zingibar)**,** (7b) Abyan (Batis)**,** (8) Mukeiras**,** (9a) Lawdar, (9b) Dathina, (10) Al-Wadhee, (11) Shabwa Ataq, (12) Shabwa (Haban), (13) Hadhramaut (Syoon, Tream), (14) Hadhramout (Mukalaa), (15a) Al-Mahrah (Qeedhah), (15b) Al-Mahrah (Haoof). * endemic to Yemen, ** endemic to Arbian Peninsula

## Results and discussions

### General data

The elderly informants were the main reference for traditional knowledge, and the young showed a weak of information. Local people in southern Yemen use traditionally wild plants as natural sources for medicines and food, as well as for a variety of other purposes. This study reports the ethnobotanical data of wild plants from southern Yemen concerning traditional domestically and other non-medicinal non-food uses of plants.

The survey identified 73 wild plant species belonging to 28 family with various local traditional uses (Table 2). The most dominant wild plants belong to Fabaceae (14 species) mostly *Acacia* species, which are of particular importance as constructions materials and as forage for livestock and beekeeping. This is followed by Asteraceae (6 species), Malvaceae (5), Apocynaceae (4) and Moraceae (4) (Table 2). Most used plants are trees (27 species) followed by shrubs (24 species), herb (19), and grass (2) and one plant as a climber. While the most common useful parts of plants were reported as stem (37 species), leaf (30 species), wood (27), flower (12), entire plant (16), fruit (8), bark (7), gum (2), latex (2) and liquid exudate (1). Various wild plant species have high abundance in the flora of the plant study areas, showed importance for the environment and people. The number of wild plants in southern Yemen studied is more than the number of wild plants used as edible food plants [8] but much less than those used in traditional medicines [7].

### Distribution of the plant species

*Ocimum forsskalii* is endemic to southern Yemen. Two reported plants are endemic to southern Yemen and west of Oman: *Cynanchum vanlessenii,* and *Vachellia yemenensis*. *Ziziphus leucodermis* and *Boscia arabica*. Species endemic to Arabian peninsula are *Aloe sabaea* and *Dracaena serrulata*. while *Euphorbia cactus* is endemic to Arabian Peninsula and islands of Kanari. Many species were determined to have distribution only in the regions of Arabian Peninsula, and African Horne, these include: *Cyphostemma digitatum, Euryops arabicus, Grewia tembensis, Grewia trichocarpa, Indigofera arabica, Kleinia odora, Searsia retinorrhoea* and *Senegalia asak* [7].

### IUCN STATUS of the threatened plants

From reported plants, some species have been enlisted in the IUCN (conservation status) Red List of Threatened Species. According to IUCN STATUS, *Dracaena serrulata* was enlisted as (EN) endangered species and *Boscia arabica* has been classified in VU (Vulnerable) group*.* Twelve species have been assessed in less threatened group described as Least Concern (LC), these include *Ficus cordata, Ficus palmata, Ficus vasta, Grewia tenax, Hyphaene thebaica, Lycium shawii, Salvadora persica, Senegalia mellifera, Tamarix nilotica, Tarchonanthus camphoratus, Vachellia seyal* and *Ziziphus spina-christi.* Despite the widespread spread of these plants within the country, they face many dangers, the most important of which is excessive, irrational consumption, in addition to urban sprawl in rural areas, which threatens large areas of these plants, in addition to the lack or absence of rain in the southern regions of Yemen.

### Categories of traditional uses

These 29 local wild plant species were classified into nine main classes according to the local traditional domestic uses (Table 3). The most cited five categories were construction timber (18 species with 788 citations), handicraft (26 species with 739 citations), economic plant products (17 species with 694 citations), agriculture tools (24 species with 648 citations) and beekeeping (15 species 571 citations) (Table 3). This study showed that the traditional knowledge in southern Yemen is rich and has broad purposes in directions other than the traditional uses of wild plants in medicine and food. The three categories with highest number of plants species were handicraft (29 species), agriculture tools (24 species) and animal husbandry (21 species).

**Table 3:** Categories of traditional general uses of wild plants in order to high citation number

Category of traditional uses	Subcategories (used species number)^a^	Total plant species	Citation number
Construction timber	Roofs, Ceiling, Doors, Windows, Locks, Keys	18	788
Handicraft	Fiber tools (14): rope, cordage, basket, mat, hat, pillows, Broom, Cook/Kitchen tools (12), Defense tools (4)	29	739
Economic and commercial plant products	Arabic gum (2), Charcoal (5), Ink (3), Refreshing drink (1), Fixed oil (1), Tanning agent (4), Dyestuff (5)	17	694
Agriculture tools	Tools for plowing: Wooden plow (*mehrath*) (11), Handle of sickle (*masrab*) (1), Stick to separate grains *(malbag, moher*) (7), Wooden grain measure (4), Hollowing stick (*methrah*) (1) Handle of axe (*fas*) (1), Poles (3), Fence (3)	24	648
Beekeeping	Bee: Bee forage (11), Wooden beehive (5)	15	571
Health aid	Insect repellant (7), Preservative agent (6), Cleansing (5), Tree for shade (3), Walking stick (2)	21	570
Animal husbandry	Livestock: Livestock fodder (8), Milk production (6), Livestock fence (2), Shepherd's stick (1)	21	524
Fuel	Firewood, Dried fire-herb	14	135
Ornamental		4	106

^a^Some species used for more one traditional use

### Wild plants used as source for traditional domestic handicraft work

The first category is the wild plants that are used as natural sources for traditional domestic handicrafts (29 species with 739 citations); this includes fibers tools: rope, cordage, basket, mat, hat, pillows, broom, (14 species), cook and kitchen tools (12 species), broom and defense tools (4 species) (Tables 2, 3).

#### Fibers

**Fig. 2:**
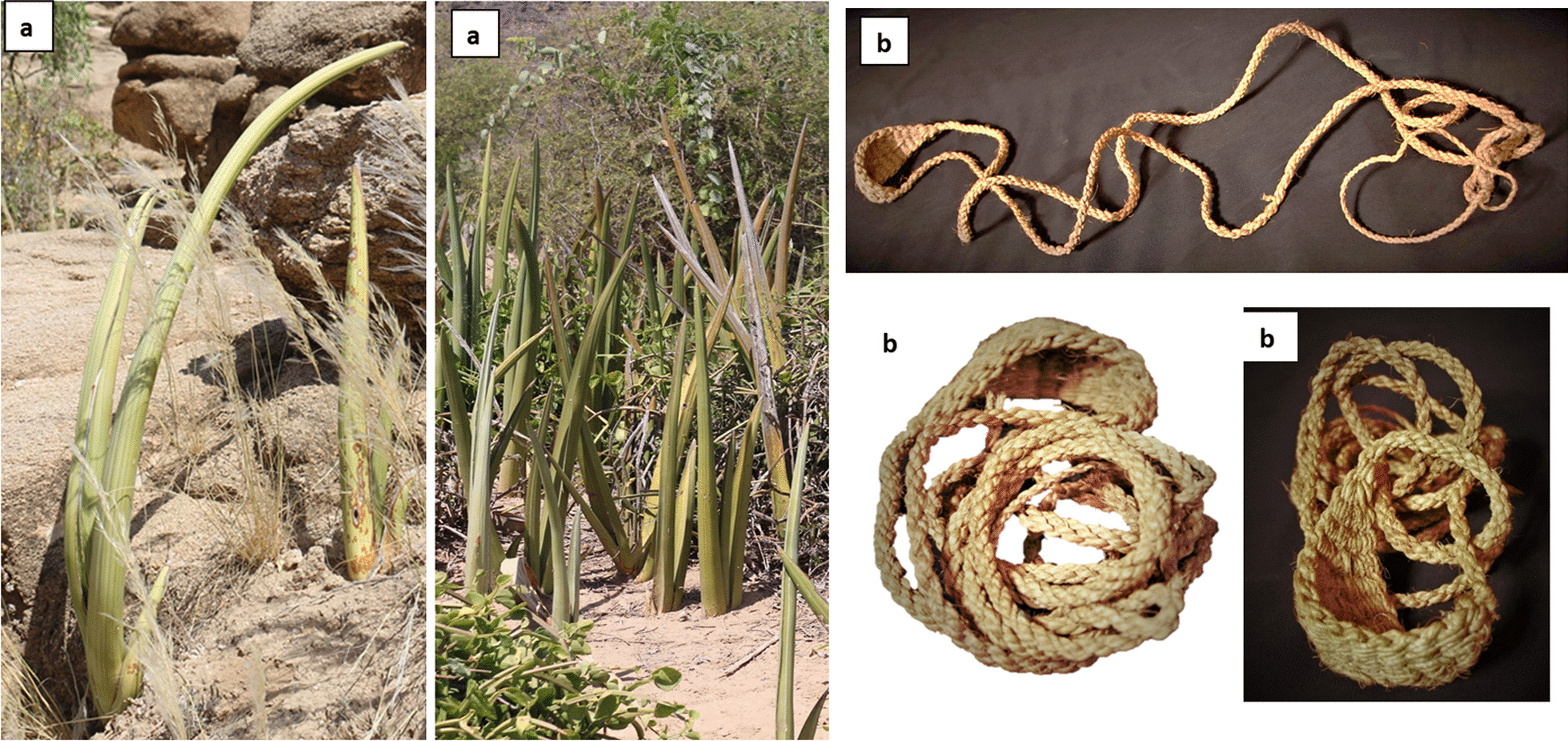
**a** Wild plant as a natural source for fibers used traditionally in southern Yemen, its scientific name *Dracaena hanningtonii* (*Sansevieria ehrenbergii*); it is locally called "*seni*". **b** Traditional sling named locally "*walaf* or *wadhaf*"; it is prepared traditionally from fibers of leaves  of *Dracaena hanningtonii.* The photos were taken by the author

*Dracaena hanningtonii* (syn. *Sansevieria ehrenbergii*) (*seni*) is the most common local wild plant that used traditionally for obtaining fiber for making traditional handicraft of rope *"habl"* and cordage *"habl",* and slings thin cord *"walaf"* (Fig. 2). The process of making fiber is called locally (*sehieg*); where the leaves are loosened by hit or rubbing with a sharp stone to remove the epidermis of leaf and to get the fibers and wrapped twisted on the thigh for weaving and making thin ropes (*sehieg*) that is sling-like. Th sling is the famous traditional product obtained from the fibers of the leaves of *D. hanningtonii* to protect crops from birds; it is named locally “*walaf, wadhaf*” (Fig. 2). This sling is used by local people for throwing small stones to expel birds away from the agriculture field protect their crops, especially wheat, from attack of these birds. Such traditional slings are known as weapon in indigenous traditional knowledge in South American [33]. We observed that there is no difference between the forms of both slings made by local people in southern Yemen and by Native Americans. *D. hanningtonii* is used as a source of natural fibers and traditional rope making in other tropical regions such as Tanzania and Ethiopia [18, 34, 35]. Its leaves fiber contained cellulose, hemicellulose and lignin; this natural cellulose showed a tensile strength of 50–585 MPa [36].

In addition, the leaves of *Dracaena forskaliana (Sansevieria forskaliana*) are used as fiber source in case of lack of *D. hanningtonii* (*S. ehrenbergii*)*.* In addition, barks of some plants can be used as fiber sources for making cordage and ropes such as inner barks of *Ficus cordata* and *Ficus vasta*, while less use was reported for bark of *Calotropis procera* and *Hyparrhenia hirta*. Other fibers can be used to make baskets, rope and cord from the leaves of *Dracaena serrulata* and *Hyphaene thebaica* and *Typha domingensis. Dracaena serrulata* was reported in Oman for rope making [17].

**Fig. 3:**
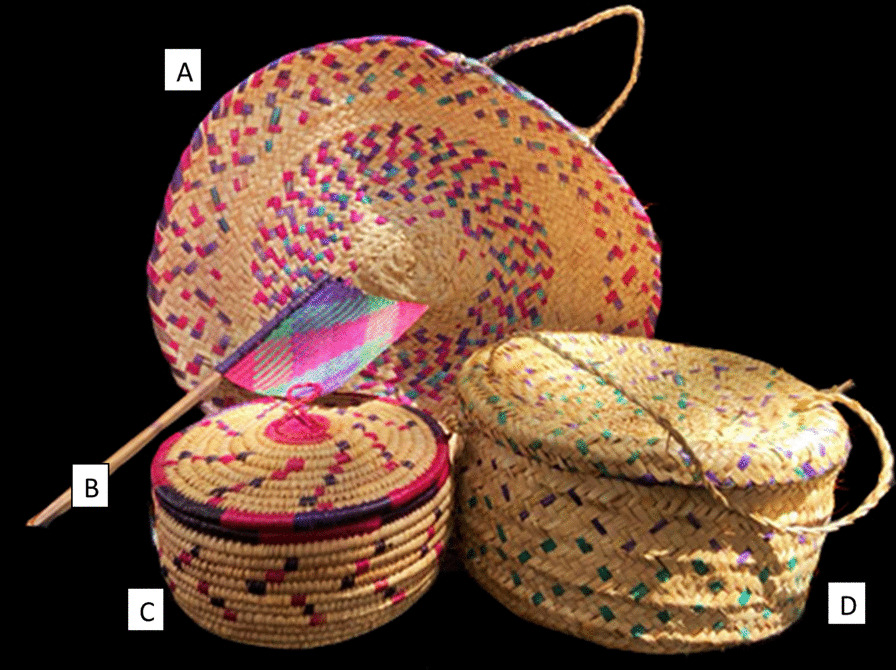
Handicraft made from the plant fibers. **a** Dining mat, **b** hand fan, **c**, **d** pot for storing bread**.** The photo was taken by the author

On the other hand, the cultivated palm tree *Phoenix dactylifera* L. (Arecaceae) (*nakhil*) is considered one of the most important cultivated plants used locally for various purposes, including the use of its leaves (*azaf*) in the manufacture of mat (*haseerh*), basket (*sellah*), hand fans (*marwaha*), hat (*qubaah*), ropes (*single: "habl"; plural" hibal*) and bed stead (*qaadah*) (Fig. 3). The date palm is the most used tree in Arabian Peninsula countries to make these traditional tools such in Oman [17]. Generally, the date palm tree has been famous in the countries of the Arab world since the dawn of history as a source of food and medicine (fruits), handicrafts used both domestically and abroad (leaf fibers) and construction (wood).

#### Pillow filling

The fruits (diaspores) of *Aerva lanata* and *Aerva javanica* are usually used for making pad for sleep and for filling pillows and saddle of donkey in study origins. The same use of *A. javanica* as a natural material in pillow stuffing is also found in Pakistan and Oman [17, 37]. Fruits of *Tarchonanthus camphoratus* used for Pillow filling but with less citation.

The results showed that there are three types of fibers documented in the study according to the used parts: leaf fibers, bark fibers and fruits (diaspores) fibers. It is said that the leaf fibers of *Dracaena hanningtonii* are the hardest fibers. In addition to wild sources of fibers, the fruits of *Gossypium hirstum* are cultivated in Zingibar (Abyan) that gives most important cotton fibers in the last seven decades.

#### Cook and kitchen tools

Woods of *Vachellia nilotica* (*Acacia nilotica*)*, Vachellia etbaica* (*Acacia etbaica*)*, Vachellia seyal* (*Acacia seyal*)*, Senegalia hamulosa (Acacia hamulosa*), *Commiphora gileadensis* and *Tamarix nilotica* are locally used to make wooden cup, wooden plate and wooden pestle for crushing vegetables such as onions and garlic.

Stems of *Vachellia gerrardii* (*Acacia gerrardii*), *Senegalia asak (Acacia asak)*, *Cordia quercifolia*, *Olea europaea* subsp. *cuspidata* and *Grewia tembensis* were reported to use as solid long sticks after removing the outer barks of the stems. This prepared stick is used by hand as manual wooden mixer (*medhrar; mahwasch*), to mix flour Mel (*taheen*) with boiling water during cooking. The common food made by this method is called locally (*zad*); this traditional food is served in a special wooden bowl (*kuaadah*) with hardness character and a smooth surface which made of wood from *Ziziphus spina-christi* or *Vachellia nilotica*. It is said that many people in the countryside still prefer to eat the specific meal (*zad*) in this wooden bowl because it gives a.

#### House cleaning tools (broom)

The whole herb of *Pulicaria undulata* and *Euryops arabicus* and the leaves of *Schoenoxiphium sparteum*, and *Hyphaene thebaica* were reported to use as traditional brooms for house cleaning. Dried herb *P. undulata* of is an aromatic plant therefore, after cleaning; it spreads aromatic smell in the house.

In Europe, other plants are used to make traditional brooms. Among these are plants of the genus *Artemisia*, which gives an aromatic scent after sweeping [38], while plants of the genus *Pulcaria* are used in southern Yemen.

Commercially, these traditional brooms are made from the palm tree, especially in Hadhramaut and Lahj. The manufacture of traditional brooms from local plants helps many poor people, especially women, as a source of income for the family, as is the case in other world regions such in south Africa [39].

#### Defense tools

In the past, the local people in some study areas have used the ashes of sticks of *Calotropis procera* to mix with sulfur and salt to make “gunpowder-like”. The bark of *Ficus cordata* is used to make a wick for setting fire to ancient guns. Stick from *Cordia quercifolia* is used as defense tool called (*assa*, *mohwer*). *Buxus sempervirens* was the first plant from which humans made pointed sticks for protection or for lighting fires. It was a 171,000 years ago in the late Pleistocene era, when wooden tools were found at an archaeological site in Italy [40].

The manufacture of handicrafts from plants is similar to neighboring Arab countries, such as Oman, where handicrafts are widespread among rural people, mostly in both countries, and they are distinguished by high skills. They are still widespread in Yemen, but in Oman and the Gulf countries, the handicraft profession declining due to dependence on the petroleum industries [20].

### Wild plant used for making agriculture tools

The second category is plant used for making agriculture tools with 24 species with 648 citations. This category is classified into tools for plowing: wooden plow (*mehrath*) (11 species), harvesting tools (8), wooden grain measure (4), handle of axe (*fas*) (1), poles (3) and fence (3) (Tables 2, 3).

In southern Yemen, agriculture often depends on torrents of rain. It also relies on traditional wells that were dug by ancestors with traditional tools, but in the past three years, the construction of dams in the valleys and the digging of tubewells has introduced. Changes in climate and the increase in temperature have led to a decrease in the level of rainfall in southern Yemen, which rarely falls, especially in the southern regions, and devastating floods often occur. This scarcity of rain has led to water scarcity and thus a lack of agriculture and livestock production, and water no longer reaches the population in cities on a regular basis. Such arid tropical climatic conditions affecting agriculture also exist in neighboring countries [41].

The development of traditional agricultural tools and thus agricultural methods among the Yemenis since the beginning of the history of agriculture in Yemen led to the purification and selection of crop varieties that doubled during the pre-Islamic and medieval eras such as the number of wheat and sorghum verities [42].

In the countries of the Arabian Peninsula, which are rich in petroleum resources, the craftsmanship of handicrafts has declined greatly due to the exploitation of oil resources in industry and due to the increasing import of goods [43]. Conversely, in Yemen, which is still in economic weakness, these crafts are still continuing among the people of the countryside to cover the internal needs of popular handicrafts.

However, most population of Yemen are living in the rural areas and are poor. Therefore, there is a continuing increase in the dependence of poor people on natural rural resources of livestock, fuel wood, crops and fodders.

#### The traditional plow (mehrath)

The traditional agriculture knowledge in southern Yemen has ancient history including traditional soil tillage system. The traditional wooden plow (traditional plough) (*mehrath*) is pulled by two oxen (*dhamed*). Its components are the share (*sehb*) made up of steel and the main handle made up of *Acacia* wood*.* The handle and the beam, which connected to a wooden yoke, hanged on the necks of two oxen (*dhamed*) that pull the plow during tillage of the soil. The handle is made from woods of *Acacia* species including *Vachellia nilotica, Senegalia hamulosa, Senegalia mellifera* and *Senegalia asak.* In addition, wooden plow (*mehrath*) is made from wood of other preferred species such as *Ziziphus spina-christi, Balanites aegyptiaca, Commiphora kataf, Cordia quercifolia, Ficus sycomorus*, *Ficus cordata*. The yoke (*heig*) is made up of woods *Commiphora gileadensis*, *C. kataf* and *Cordia quercifolia*. It is said that these used woods make plowing easy and facilitate the tillage process, because they are light and durable woods. The stem of *Calotropis procera* was reported to use as hollowing stick as wooden tube (*medhrah*) connected to the wooden plow (*mehrath*) to pour and distribute the seeds during tillage. The traditional plowing (*mehrath*) aids for conservation of agriculture in southern Yemen since the ancient times. The inherited traditions of using natural sources remain the safest in the face of mechanical industrial intervention affecting the environment, such as the traditional use of agriculture [44].

The ancient agricultural terms and farming techniques in Yemen since pre-Islamic history that were revealed in the archaeological inscriptions have not changed much in the local language until today [16]. Likewise, post-Islamic agricultural reforms found in the medieval Arabic manuscripts are still used by the population in the rural origins in Yemen, for example the Arabic local name for plough " *mihrath*" or "*mehrath*" and described the Yemeni usage of plowed furrow [45].

#### Harvesting tools

The handle of sickle (*masrab*) is made up of wood *Vachellia nilotica* for harvesting process for crops, cereals and grasses. To separate the grains from the ears of the corns, a solid stick from the stems of wild plants can be used as a beater (stick). This wooden grain separator is locally called (*malbag or moher).* The bark is removed from stem that used as stick (*moher*). It is made up of wood from selected plants including *Grewia tenax, Grewia tembensis, Grewia trichocarpa, Senegalia asak*, *Vachellia yemenensis*, *Lycium shawii* or *Ehretia cymosa*. The farmers use the wooden separator to separate the grains by beating the ears of the cereal harvested crop. Similar uses for other species of *Ehretia* (*E. btusifolia*) was reported was found to use for making same agriculture tools in Pakistan [19]. To remove impurities from grains of crops, the herb of *Pulicaria undulata* is used for sweeping these unwanted materials from grains.

#### Wooden measuring tool

A wooden bowl is locally called (*mekyal*); it is a wooden grain measure that is used as volume unit of measure for grains of cereals, coffee fruit, and sesame seeds. It and made up of the wood of *Commiphora kataf, Commiphora gileadensis* and *Ficus sycomorus.* Wood is hard yet light. This facilitates the process of filling the bowl and pouring its content during the process of weighing.

#### Wooden ax (fas) and shovel (magrafah)

The handle of the wooden ax (*fas*) is made up of stem of *Grewia tenax* and *Searsia retinorrhoea*. Stem of *Grewia tembensis* can be used to make wooden handle of hoe (*magrafah*), which is used to dig the soil manually. In Sudan, *G. tenax* wood was reported as common source for making agricultural tools [46]. Documenting agricultural methods and their development, such as the plow, sickle and axe, in ancient societies facilitates knowledge of the dynamics of agricultural development in the society [47].

#### Trees as Fencing for field

The trees of *Ziziphus spina-christi* are planted as hedge for protect agriculture fields. The view of the farms and fields in the countryside of southern Yemen is familiar with an ocean of zissovo trees, which carry the crops from the wind and provide suitable shade for them and for the farmers and shepherds. Stems of *Ficus cordata* are used to make fences to protect the coffee, corn and millet plants.

*Tamarix nilotica* works to repel wind and sand. In southern Yemen, other cultivated and introduced plants were planted to protect against the wind and also to improve the tropical climate since thirty years ago, these include introduced plants *Conocarpus lancifolius* in Aden [48]. *Parkinsonia aculeata*, *Prosopis juliflora, Prosopis farcta* and *Prosopis cineraria* between Aden and Hadhramout,

#### Wood aiding in drawing water from the well

Stems of *Ziziphus spina-christi*, *Vachellia nilotica* and *Vachellia etbaica* were reported to use for building wooden poles on the well to draw water in a bucket.

### Wild plant used as health care aids

This third category deals with 21 plants species with 570 citations; it contains some subcategories including insect repellant (7 species), preservative agent (6 species), cleansing (5), shade tree (3) and walking stick (2) (Tables 2, 3). The traditional uses of many wild plants were reported in the field of general health care of human and animal.

#### Insect repellant and fumigant

The dried stem of *Cynanchum viminale* subsp. *stipitaceum* (*Sarcostemma viminale* subsp. *stipitaceum*) is burned as fumigant to use as incense (fumigant); it is said it makes healthy air after childbirth and used also for cloths of new births. In addition, the dried leaves of *Aloe inermis* and *Aloe vacillans* are used as fumigant to repellant insect during childbirth. On the other hand, *Aloe trichosantha* is traditionally used in Ethiopia to repel insects [49].

Seven plants were reported to repellant insects including *Aloe inermis, Aloe vacillans, Artemisia abyssinica, Blepharis ciliaris, Cynanchum vanlessenii* and *Cynanchum viminale.* The squeezed liquid of the fresh leaves of *Blepharis ciliaris* is sprayed at home to kill fleas. Stems of *Cynanchum vanlessenii* and *Cynanchum viminale* were burned as fumigant to repellant the insects in livestock housing. The herb of *Artemisia abyssinica* was placed between the clothes to repel insects. In addition, the essential oil from *Artemisia absinthium* has been proven to be insect repellant [50].

#### Preservative agents

The powder of the leaves of *Vachellia nilotica* (*Acacia nilotica)* is traditionally added in small amount in drink water container as preservative agents (*qaral*). This local traditional use has been confirmed in our previous study that identified its strong antimicrobial and antioxidant activities [51]. Woods of some plants can be used as container for drink water for livestock such as wood of *Vachellia etbaica* (*Acacia etbaica*) and *Ficus cordata, Commiphora gileadensis* where a rectangular cavity is made in the stem of the plant to store water. It is said that these stems preventing the attacks of insect and the microbial contamination. Fresh raw herb of *Indigofera arabica* is rubbed on the wooden pots, followed by burning part of the herb; it is said that its fumigant has activity against microbial contamination therefor it used as preservative for drink water or food. Fresh herb of *Artemisia abyssinica* is cut in small pieces, and it is distributed in rooms and between document papers, to preserve against insect attack. The fumigant of the dried juice of *Kleinia odora* is used as incense after childbirth against attack the microbes. In Ethiopia, this plant is traditionally used to expel insects [49].

#### Cleansing agents

Wild plants are used as hygienic agents for human hair and body care. Some cited plant species are used as natural soap and shampoo, for washing body and hair including leaves of *Corchorus depressus, Corchorus trilocularis* and *Ziziphus spina-christi* powdered and mixed in water. This mixture is used to smear women hair for some hours and followed by washing to clean. *Ziziphus spina-christi* is the most common plant used to wash hair by women as traditional shampoo. The leaves powder of *Z. spina-christi* is mixed in water; this is called “*qasel*”; its powdered barks are mixed in little water as paste; this is called "*lagub*".

Both preparations are used as shampoo to wash and clean women hair. In addition, paste of leaves of *Tarchonanthus camphoratus* was reported to clean body as soap.

#### Shade tree

**Fig. 4:**
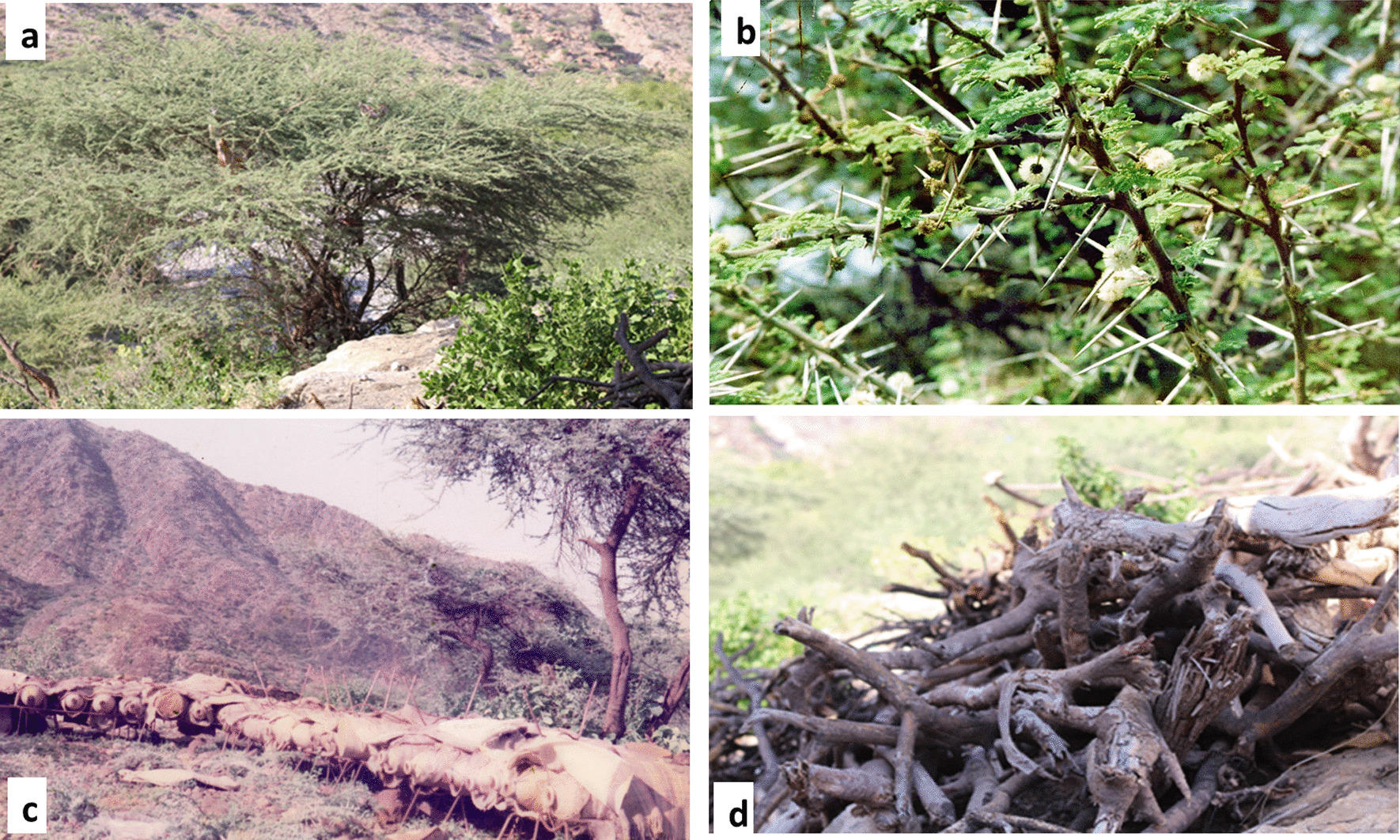
**a, b** *Vachellia tortilis* (*Acacia tortilis*) is an important plant used traditionally in southern Yemen as source for bee forages, **b** flowers of *Vachellia tortilis* **c** bee hives **d** dead stems of *Vachellia tortilis* used as good firewood and for prepared charcoal. The photos were taken by the author

In southern Yemen, the sub-tropical climate is dominant during the year [1]. Therefore, the shade of trees is an important factor to protect people and livestock from the hot sun. Two plant species widely have high abundance in each study areas and are used for shade to protect humans and livestock from the sun in the tropic areas: *Ziziphus spina-christi* and *Vachellia tortilis*. In addition, *Vachellia tortilis* grows in the arid areas during years without rain; therefore, it is the most important tree as housing and shade for livestock because of its strength and steadfastness to protect against the sun and wind (Fig. 4).

#### Walking sticks

Stems of *Grewia tenax* and *Grewia tembensis* were reported to make good and strong walking stick to help elderlies or sick persons to walk.

#### Flavoring agent

*Senegalia mellifera* barks are burn and used as fumigant into pot to get good taste and smell by storing food.

### Wild plants used as source for products with economic value

The fourth category is the economic and commercial plant products from 17 species with 694 citations. These natural products are classified as dyestuff (5), charcoal (5 species), ink (3), tanning agent (4), flavoring agent (4), Arabic gum (2), refreshing drink (1) and fixed oil (1) (Tables 2, 3). Natural products from plants have economic value that helps improve the family's income such as gum, dyes and charcoal. On the other hand, the study shows promising sources of natural materials that can be used in general industries, such as pen ink and tanning materials.

#### Dyeing

Some common wild plant species are used traditionally as dyestuffs. *Indigastrum costatum* (black color) and *Indigofera tinctoria* (dark blue color) for dyeing of hair or body of women or men. Powder of the dried leaves of each plant are mixed in water as semi-solid, smear on hair and let drying in air or under sunlight.

The leaves of *Indigofera tinctoria* (blue to black) and *Indigofera coerulea* (black) are mixed together in boiling water to make dark blue dye solution for cloths. The leaves powder of both *Indigofera tinctoria* and the cultivated *Lawsonia inermis* is used as mixture to make red dye paste for hair and clothes. The responsible natural compound for the blue color of *Indigofera tinctoria* is indigotin [52]. *Indigofera* species such as *I. tinctoria* are well known to use as traditional dyeing in old world including south Europe [53].

Dyeing is one of the traditional uses of local plants that have declined and are nearly extinct. It must be revived through blogging and educating young people in workshops. Hadhramaut is the main geographical origin of henna in Yemen where there are also many ways to use it as a coloring material for hair, hands and feet [11]. The henna trade is one of the most important commercial agricultural activities in Hadhramout Governorate.

#### Charcoal

*Vachellia tortilis* wood was reported as high cited to provide the good charcoal from Abyan and Shabwa in the study area, which is exported to other southern Yemeni areas. Alternative plant wood-making charcoal was reported for *Vachellia etbaica*. *Vachellia tortilis* is reported in other countries as the most selected Acacia species for charcoal production, such as Ethiopia and Oman [54, 55].

#### Natural ink

The juices (saps) of *Aloe vacillans* and *Aloe inermis* are mixed with the black smoke obtained from burning wood. This mixture is used as pen ink for writing that is not erasable.

#### Tanning agent

The dried leaves of *Vachellia nilotica* (*Acacia nilotica*) were high cited (100%) to use for tanning animal leather that use by local peoples in the study area until today (Table 2). Alternative wild plants were cited to use as tanning agents include leaves of *Vachellia gerrardii* and *Cyphostemma digitatum.* In addition, leaves and latex of *Calotropis procera* were cited to remove the hair of animal skin before leather tanning, mostly in Hadhramout.

Generally, the leaves of *V. nilotica* (*Acacia nilotica*) are known as rich natural source of tannins used for tanning leather [56]. In some Arabic countries including Oman and Sudan, the legume fruits of *Vachellia nilotica* (*Acacia nilotica*) are used as tanning agents; these tannins were reported to use in the manufacturing of the leather [17, 57, 58]. Barks of *Acacia mearnsii* and *Acacia xanthophloea* are traditionally used as tanning agents to make leather Tanzania [59]. Similar use for *Calotropis procera* was reported in the neighboring country Oman [17]. Recently, it was identified that the latex of the *C. procera* is safer and most suitable to the environment than the chemical agents to remove the hair of the leather [60].

#### Arabic gum

The most important Arabic gum is produced by incision of the stems of *Senegalia hamulosa* and *Vachellia nilotica*. Their dried liquid exudates form high valuable Arabic gum. Generally, the Arabic gum is well for its high value as pharmaceutical aid for drug forms such as tablet and suspension; moreover, it shows also antimicrobial activities [61]. As a result of nature's dependence in Yemen on rain only, the spread of acacia plants is small compared to Sudan, which is considered an important geographical origin for the trade in gum Arabic; therefore, the income from gum Arabic is high for the people who work in its production in Sudan [62], due to the importance of Gummi Arabica in the manufacture of various pharmaceutical forms such as tablets and suspensions.

Generally, *Acacia* species are considered an important source of income for families in rural areas in various tropical regions. They are a source of production of construction wood, gum, tannin, firewood and fodder for livestock [63].

#### Refreshing drink

The stem of *Hyphaene thebaica* produces liquid exudate by incision of the stem that is traditionally used as a refreshing, and relaxing drink with sour taste. This juice of stem was not biological investigated, while in the fruit many bioactive compounds were identified including saponins, coumarins, hydroxyl cinnamates, essential oils and flavonoids. In addition, the fruit of the plant contains sugars, protein, fats and minerals [64].

#### Fixed oils

From seeds of the wild plant *Ziziphus spina-christi*, the fixed oil is extracted*.* This fixed oil has become very popular nowadays and is sold by young people and children in the countryside. It is used for many medicinal and food purposes [7, 8]. On the other hand, sesame oil is used from the sesame plant (*Sesamum indicum*) widely grown in the countryside as a basic food and traditional medicine and for commercial purposes that help generate income for farmers.

### Wild plants used as construction (timber) materials

The fifth category cited with 18 wild plant species with 788 citations for the construction (timber) including roofs, ceiling, doors, windows, locks and keys. The important timber (wood) obtained from many local plants was reported to use for construction by local people in southern Yemen. The construction was known to the local people since ancient times with materials of stones or from clay mixed with livestock dung, often from cows, but the ceilings were carried and supported by wood, and the workmanship of doors and windows was made of tree wood.

For making of room roof timber (*gobb* or *gobba*) or making wooden boards supporting ceiling, doors (*saddah*), locks and windows were reported to use the woods of *Balanites aegyptiacus* and *Ziziphus spina-christi*, *Vachellia nilotica*, *Vachellia gerrardii, Vachellia tortilis, Boscia arabica, Ficus palmata, Ficus sycomorus, Ficus vasta* and *Olea europaea.* These are hardwoods with highest quality. In addition, sticks of the shrub *Anisotes trisulcus* were used as support materials (Roof thatching) between woods of roofs. In rare cases, the stems of *Aloe sabaea* are dried, collected and tied together for making doors. Dried powdered stems and leaves *Tamarix nilotica* and *Tamarix aphylla* are mixed with water and soil to use for home ceiling. It was said that these woods, stems and sticks used traditionally from these local plants for construction, have resistance against attack of insects, especially termites and wood mite due to moisture resistance and have strong hardness that does not break or absorb water easily. We found some old houses whose roofs were carried by wood from more than two centuries ago. The wood of *A. nioltica* is used in Pakistan and Ethiopia for same traditional uses in construction and agriculture tools [18, 19]. The woods of other species of *Acacia* are used in traditional house building in Ethiopia, including *A. mellifera*, *A. Senegal*, *A. oerfota* and *A. seyal* [18]. The wood of *Vachellia (Acacia)* species has been reported to use for construction of boat in ancient Egyptian [65].

Since ancient times, Yemeni people have been characterized by high local artistic skills in building with stones, clay and the use of wood. This produced a distinctive art of wood carpentry among the local people until today [66].

### Wild plants used as source for animal husbandry

The sixth category is the animal products process, mostly forage for livestock and their products that have 21 used plants with 524 citations, including livestock fodder (8) species, milk production (6) and livestock fence (2) (Tables 2, 3). The tropic area has high distinct valued plants used as fodder for livestock and for honeybees. The traditional production from local wild plants related to the economic value is one of the things that must be preserved, many of which are actually still practiced by young people as a source of livelihood.

Grazing and livestock breeding represent a major activity for southern Yemen constituting 39.58% of the total average number of livestock in Yemen. Goat raising comes in first place in terms of animal density during the period, accounting for 53.35% of the total livestock population in Yemen, followed by sheep (30.38%), camels (3.55%) and cows (8.08%) in the year 2012 AD [67]. The animal density of southern Yemen is due to its desert nature, where most of the population works in grazing agricultural animals.

#### Forage for livestock

The indigenous people in the study area reported different *Acacia* species for livestock fodder including *Senegalia hamulosa, Senegalia mellifera*, *Vachellia gerrardi*, *Vachellia nilotica* and *Vachellia tortilis*. It is said that the leaves of *Senegalia* an *Vachellia* (*Acacia*) species are responsible for the specific delicious taste of the sheep meat, which is known in the cities markets as premium meat, especially *Vachellia tortilis* leaves and fruits which are most grazed by livestock and have high abundance everywhere in southern Yemen. In addition, livestock eagerly eat the fruits of *Vachellia tortilis* (*A. tortilis*), and it is said that the fruits have a stimulating effect on mating of livestock especially goat. It has a highest resistance among the local plants to survival in dry seasons. It is said that the herbs of *Euphorbia inaequilatera* and *Euphorbia granulata* increase the milk of livestock. Livestock intensively graze many herbs that grow in rainy seasons; these include *Amaranthus graecizans, Corchorus trilocularis, Cynodon dactylon, Cyperus rotundus, Lactuca serriola* and *Portulaca oleracea*. In the lack of rain, livestock eat such these plants *Cissus rotundifolia* (leaf)*, Euphorbia granulat* (herb), *Euphorbia serpens* (herb)*, Lycium shawii* (leaf) and *Tribulus terrestris* (herb).

Some studies prove that *Acacia cyanophylla* foliage decreases the milk fat of livestock such as ewes [68]. Acacia is considered the most important source of fodder for livestock in many countries such as Australia [69].

#### Fence and housing for animals

*Vachellia tortilis* (*Acacia tortilis*) and *Ziziphus spina-christi* are also a plant with sharp thorns that help protection as fences for livestock. Wooden beehives are made from stems and woods of *Vachellia gerrardii* and *Dracaena serrulata* and from *Ficus* species including *Ficus vasta, Ficus cordata* subsp. *salicifolia* and *Ficus sycomorus.*

#### Milk production

Seeds of *Solanum lasiocarpum* are mixed with fresh milk of goats or sheep to become clot with delicious taste, after removing of seeds. Similar traditional use of berries of many different *Solanum* species as milk-clotting agents was reported in Africa [70]. In addition, the bark powder of *Ficus cordata* assists goat milk to become clot. A protease (ficin) was identified as responsible constituent of *Ficus palmata* latex for the clotting of the fresh milk [71]. Two drops of the fruit latex of *Ficus palmata* are added to the fresh goat milk (*labah*) turns into buttermilk (*dahen*).

On the other hand, the shell of dry fruit of the cultivated *Cucurbita pepo* L. (Cucurbitaceae) (*qaraa*) is hard outer covering that is used as container to preserve the livestock milk; it is also used to shake the milk to prepare butter.

### Wild plants used as source for beekeeping

The seventh category has 15 plant species with 571 citations. It contains bee forage (11 species), wooden beehive (5). The importance of beekeeping for the local population is that it provides work for the youth. Honey production is of great economic importance due to the fame of southern Yemeni honey from *Vachellia (Acacia)* species and *Ziziphus* species. Of them, two species were reported as the most important specific bee's forage: flowers of *Ziziphus spina-christi* and *Vachellia tortilis* (Fig. 4). The first honey type with high valuable quality that is available in the Yemeni markets is called locally (*asal ilb*) originating from flowers of *Ziziphus spina-christi* (*ilb*) with high nutritional value and distinctive taste. These types of honey from southern Yemen are exported to neighboring countries in the Gulf and the Arabian Peninsula. It is said this honey has a distinctive taste and has great health and nutritional benefits. The honey from Iran obtained from the species of *Ziziphus* was reported to have high nutritional value and medicinal properties such as high antimicrobial activities [72]. The second premium honey known in southern Yemen is called (*asal somer*) obtained from the flowers of *Vachellia tortilis* (Fig. 4)*.* Flora of southern Yemen showed high diversity with important nutritious forage for honeybees, including *Vachellia* and *Senegalia* (*Acacia*) species such as *Vachellia nilotica, Senegalia mellifera* and *Vachellia gerrardii* and *Senegalia asak,* besides other genera species such as *Anisotes trisulcus*. In addition, honeybees are attracted to the flowers of *Aloe inermis, Cynanchum viminale* and *Euphorbia cactus* with pungent taste. Flowers of *Ziziphus spina-christi, Vachellia nilotica* and *Anisotes trisulcus* were reported as major bee forage sources in Saudi Arabia, and flowers of *Ziziphus spina-christi* and *Vachellia tortilis* are the major bee foraging [73–75], both countries have similar flora and arid geography to southern Yemen.

The high quality of nutrition from wild indigenous plants for livestock and bees makes meat and honey have a distinctive taste and a high nutritional value used frequently among the population of the country, and even it is requested from the neighboring Arab countries. The best types of honey in the country are from Abyan, Shabwa and Hadhramout. The best type of honey is called "*Doaani*" honey from flower of *Ziziphus spina-christi*, where special types of yellow and black bees that are resistant to drought have been identified *Apis mellifera yemenitica* [76]*.* This honey from *Z. spina-christi* is also called *"sidr* honey" in other regions of the country and is characterized by high quality [77].

Beekeeping and honey production is an ancient tradition in southern Yemen as well as the region in the Arabian Peninsula. The difference in the quality and taste of honey is due to the difference in flora between the countries of the world. In general, the tropical flora, especially flowers of *Acacia* species, gives off a distinctive quality of honey. Beekeeping and honey production have been known since ancient times, as local residents knew where beehives were located in the mountains and valleys, and today they make beehives from tree trunks (logs). Honey production ranks fourth in the local production economy [76].

During the past two decades, beekeeping has expanded, especially among young people who depend on migration of colonies from pasture to pasture between the country’s governorates, where there is rain and flowering time of the plants. Beekeeping has become one of the most important professions that generate good profits and provide work for a large sector of young people. Perhaps due to the lack of job opportunities due to the ongoing war, young men and men found beekeeping as a source of income for their living. During that period, a generation of residents acquired high skills in raising and producing the finest types of honey, which were then exported abroad. Beekeeping workers have great knowledge and traditional experience in handling bees, beehives and building bee houses and in determining the flowering dates of plants in various regions of the country, and they travel from region to region carrying bee houses in special transport vehicles.

### Wild plants used as source for fuel

The eighth category was cited with 14 wild plant species with 135 citations. It is the firewood sources. Wood in wild forest areas was of great importance in human life until the last century as the only local source of energy. Therefore, the agricultural areas were strongly protected by each tribe. *Acacia* species are common important sources for firewood due to large distribution abundance in the study area. The stems of *Vachellia* and *Senegalia* species have high quality of firewood include *Senegalia asak, Vachellia flava, Vachellia gerrardii, Vachellia tortilis* and *Senegalia mellifera.* Other plants species were reported to use as firewood such as *Commiphora kataf, Commiphora gileadensis, Grewia tenax, Grewia tembensis, Grewia trichocarpa*, *Tamarix nilotica, Tamarix aphylla*, *Anisotes trisulcus*, *Breonadia salicina* and *Ficus cordata.* The dried herb of *Fagonia indica* as thatch was reported as very flammable herb that helps start burning in large, hard firewood. Generally, the dead trees are used as suitable and common firewood (Fig. 4).

In ancient archaeological stoves (hearth) from Yemen, the presence of fuel remains of the *Acacia* sp, *Ficus* sp., *Tamarix* sp. and *Ziziphus* sp. was discovered, which indicates its use in Yemen as a fuel material 8000 years ago [78]. These are still used today as fuel except *Ziziphus* sp.

### Wild plants used as ornamental plants

The ninth category is the less cited category with four ornamental wild plants with 1o6 citations. wild common ornamental plants are *Artemisia abyssinica* (*ozab*) and *Ocimum forskolei* (*schuqur*) that are used as houseplant ornamental. *Aerva lanata* and *Aloe vacillans* are *used as* decorative plants on graves. In addition, many of the plants cultivated are used as ornamental plants, including *Artemisia arborescens* (Vaill.) L. Asteraceae (*ozab*). The cultivated ornamental species include *Ocimum basilicum* L. (Lamiaceae) (*schuqur, shahadah*) and *Artemisia arborescens* (Vaill.) L. (Asteraceae) (*schuqur-abiadh*).

### Most cited used plants

The highest cited plants are three local plants: *Ziziphus spina-christi* (514) *Vachellia tortilis* (459) *Vachellia nilotica* (378); each of them is used for many different purposes in the daily life of local people, in construction, agriculture, handicraft, livestock husbandry, beekeeping and even in economic production beneficial to families (Table 4). These three plants are the most important features and characteristics of the flora of southern Yemen.

**Table 4:** Twenty top most important traditional useful wild plants cited by most informants

Name of plant (total citation number for different uses)	High citation number for most individual traditional use
*Ziziphus spina-christi* (514)	Beekeeping (102); Construction timber (100); Agriculture tool (88); Health aid: Cleansing, leaves used as shampoo (100)
*Vachellia tortilis* (459)	Beekeeping Bee forage (100); Animal husbandry; Livestock fodder (100); Economic value: Wood provides good charcoal (100)
*Vachellia nilotica* (378)	Economic value: Tanning agent (100); Construction timber (87); Agriculture tools (63); Handicraft: Cook /Kitchen tools (54); Arabic gum (50)
*Anisotes trisulcus* (173)	Construction timber (100)
*Dracaena hanningtonii* (100)	Handicraft: (Fibers) rope (100)
*Aerva javanica* (100)	Handicraft: Fiber: Pillow filling (100)
*Vachellia gerrardii* (98)	Construction timber (45)
*Calotropis procera* (72)	Economic value: Tanning agent (50)
*Ficus cordata* (98)	Agriculture tools (52)
*Pulicaria undulata* (82)	Handicraft: House cleaning tools (57)
*Indigofera tinctoria* (80)	Economic value: Dyestuff (80)
*Cordia quercifolia* (99)	Construction timber: Wood for the ceiling (50)
*Balanites aegyptiaca* (78)	Agriculture tools: (78)
*Lycium shawii* (78)	Agriculture tools: (78)
*Senegalia asak* (77)	Beekeeping: Bee forage: (50)
*Senegalia mellifera* (97)	Beekeeping: Bee forage: (55)
*Vachellia etbaica* (87)	Handicraft: Cook/Kitchen tools (50)
*Tamarix nilotica* (98)	Construction timber (47)
*Artemisia abyssinica* (70)	Houseplant ornamental (50)
*Boscia arabica* (65)	Construction timber (65)

## Conclusions

In addition to being used as medicines and food, there are wild plants used for other non-medicinal and non-nutritional that are important resources for other purposes in human life. However, the ethnobotany of medicinal and food plants in Arabic countries has rarely been studied, while this field of non-medicinal and non-food plants has not been studied before including Yemen. This rarely studied field needs more studies and documentation as an important human heritage and to preserve it from extinction; therefore, this study completes the third part of the ethnobotany documentation of southern Yemen (non-medicinal and non-food uses); it contributes to the documentation and preservation of this traditional knowledge in the general uses of wild plants, including traditional crafts as part of the ethnobotany of southern Yemen. It was found that the traditional knowledge of wild plants in southern Yemen is not only a source of medicine and food, but also for preparing traditional tools and means that benefit local people in their daily lives.

The highest cited used plants were *Ziziphus spina-christi*, and *Vachellia tortilis (Acacia tortilis).* These two plants received the highest citations for many traditional uses in various fields, such as making construction and agricultural tools (wood), fodder for livestock (leaves and fruits), food for bees (flowers) and firewood (dead wood and sticks). The trees themselves are also considered an important shade for farmers, shepherds and livestock. These two plants give a distinctive and noticeable appearance in the nature of the flora of southern Yemen. This is followed by the high importance of citations for other many species of the *Acacia* genus that are widespread in the region.

Local traditional tools have historical and economic significance for generations to know how the ancestors including shepherds, farmers, traditional construction workers and indigenous people in the countryside were using local sources and local wild plants for their daily life without relying on import of industrial products and materials from abroad. Therefore, documenting these natural sources is important to create a developed local industry from local resources in future, especially for developing countries.

The challenges of nature and the environment toward the daily life of local people are led to find natural materials from wild plants for food, medicine and other general useful aspects of daily life. This traditional three-sided use is still present and used in rural areas and even somewhat used in various cities. This study indicated that the indigenous people of southern Yemen have high valuable traditional knowledge on wild plants due to a strong relationship with nature. This strong relationship is evidenced by the protection of the tribal traditions of plants in the rural areas as a cultural heritage that is respected by the local population, so there is no negative impact on the plant populations by the local people.

The study revealed, documented and analyzed for the first time the traditional indigenous knowledge inherited by the local people in the southern Yemen to make traditional tools from wild plants to use in daily life. It documented traditional methods for making traditional tools including tools of agriculture, construction, domestic handicraft and self-defense; in addition, it documented the resources of fodder for animal, livestock and bee. In the last three decades, the dependence of the local population on vegetable wood as fuel in homes has greatly decreased due to the presence of natural gas from local sources in the country. Reliance on local wood in construction has decreased completely, compared to dependence on imported wood. Grazing by livestock remained, but to a lesser extent than in the past, due to reliance on many local sources of fish and poultry. However, during the recent war in Yemen, which lasted nine years until today, it observed increasing to the use of these natural resources.

## Data Availability

All data generated or analyzed during this survey are included in this article.
